# Neuroinflammation in the normal-appearing white matter (NAWM) of the multiple sclerosis brain causes abnormalities at the nodes of Ranvier

**DOI:** 10.1371/journal.pbio.3001008

**Published:** 2020-12-14

**Authors:** Patricia Gallego-Delgado, Rachel James, Eleanor Browne, Joanna Meng, Swetha Umashankar, Li Tan, Carmen Picon, Nicholas D. Mazarakis, A. Aldo Faisal, Owain W. Howell, Richard Reynolds

**Affiliations:** 1 Department of Brain Sciences, Faculty of Medicine, Imperial College London, London, United Kingdom; 2 Department of Bioengineering, Faculty of Engineering, Imperial College London, London, United Kingdom; 3 Department of Computing, Faculty of Engineering, Imperial College London, London, United Kingdom; 4 Data Science Institute, Imperial College London, London, United Kingdom; 5 Institute of Life Sciences, Swansea University Medical School, Swansea University, Swansea, Wales; 6 Centre for Molecular Neuropathology, Lee Kong Chian School of Medicine, Nanyang Technological University, Singapore, Singapore; Max-Planck-Institut fur experimentelle Medizin, GERMANY

## Abstract

Changes to the structure of nodes of Ranvier in the normal-appearing white matter (NAWM) of multiple sclerosis (MS) brains are associated with chronic inflammation. We show that the paranodal domains in MS NAWM are longer on average than control, with Kv1.2 channels dislocated into the paranode. These pathological features are reproduced in a model of chronic meningeal inflammation generated by the injection of lentiviral vectors for the lymphotoxin-α (LTα) and interferon-γ (IFNγ) genes. We show that tumour necrosis factor (TNF), IFNγ, and glutamate can provoke paranodal elongation in cerebellar slice cultures, which could be reversed by an N-methyl-D-aspartate (NMDA) receptor blocker. When these changes were inserted into a computational model to simulate axonal conduction, a rapid decrease in velocity was observed, reaching conduction failure in small diameter axons. We suggest that glial cells activated by pro-inflammatory cytokines can produce high levels of glutamate, which triggers paranodal pathology, contributing to axonal damage and conduction deficits.

## Abstract 2

Current thinking on the mechanisms by which multiple sclerosis gives rise to cumulative neurological disability revolves largely around focal lesions of inflammation and demyelination. However, some of the debilitating symptoms, such as severe fatigue, might be better explained by a more diffuse pathology. This study shows that paranodes in the white matter become abnormal as a result of neuroinflammation, which may be the result of the action of cytokines that cause glia to release glutamate.

## Introduction

Multiple sclerosis (MS) is a neuroinflammatory disease of the central nervous system (CNS) characterised by focal and diffuse areas of inflammation, axonal degeneration and loss, demyelination, and gliosis [1]. Although the focus of MS research has for a long time been on the demyelinating lesions, neuronal damage and axonal loss are now recognised as early and persistent factors in MS pathology [2,3] and may be the best predictors of long-term neurological decline [4]. It has been suggested that activated microglia and macrophages surrounding myelinated stressed axons [3] could initiate swelling around the nodes of Ranvier, leading to mitochondrial pathology and subsequent focal axonal degeneration [5]. The lesion-free normal-appearing white matter (NAWM) in progressive MS is actually highly abnormal and contains chronically activated microglia, dysfunctional and degenerating axons, reactive astroglia, and a compromised blood–brain barrier (BBB) [6–9]. Additionally, MRI studies have shown abnormalities in NAWM regions, especially in chronic progressive patients with long disease duration [10,11].

Axonal dysfunction in the NAWM could be promoted by structural alterations at the nodes since they are critical elements in maintaining fast and efficient saltatory action potential (AP) conduction. The interaction between the Caspr1, contactin, and neurofascin 155 (Nf155) proteins and the cytoskeletal proteins, ankyrinB, and αII and βII spectrins, contributes to the formation of the paranodal junctions (PNJ) [12–15]. The intricate and tight molecular interactions between the oligodendrocyte and the axon at these myelin free points are vital for restricting movement of membrane proteins between the various nodal zones and reducing the flow of current under the myelin sheath, but also make the PNJ particularly vulnerable to immune-mediated pathological alterations. Postmortem tissue studies of MS NAWM have shown an increase in the length of Nf155-stained paranodal structures, and a partial dislocation of juxtaparanodal Kv1 channels towards the node in a proportion of axo-glial junctions [16]. However, the functional significance and mechanisms underlying the paranodal/nodal disorganisation in MS NAWM are unclear. Live laser-scanning coherent anti-Stokes Raman scattering (CARS) imaging of spinal cord myelin of rodent axon tracts exposed to elevated glutamate levels, pathological Ca^2+^ influx, and calpain1 activation, has demonstrated paranodal splitting [17–19]. Animal studies performed with conditional knockouts of the paranodal proteins Caspr1 [20], Nf155 [21], βII spectrin [22], and 4.1.B protein [23,24] showed a lack of tight septate junctions, an increased peri-axonal space, dislocation of the juxtaparanodal voltage-gated channels Kv1 towards the PNJ, and functional alterations such as motor tremors and reduced conduction velocities. Taken together, this suggests a possible model of molecular paranodal disorganisation due to Ca^2+^ accumulation mediated by glutamate activation of the N-methyl-D-aspartate (NMDA) receptors located in the cytoplasmic processes of the oligodendrocyte at the PNJ [25–28]. Multiple magnetic resonance spectroscopy (MRS) studies have demonstrated elevated glutamate levels in both acute MS lesions and NAWM tissue [29–31]. Pro-inflammatory cytokines, such as TNF, can stimulate microglia in an autocrine/paracrine manner to induce glutamate release [32,33], as well as blocking astrocytic glutamate transporters crucial for glutamate homeostasis [34–36]. Recent data from human tissue studies have shown the presence of increased levels of TNF signaling in the MS brain [37].

Here, we report that disruptions of the Caspr1 expressing PNJ structures and Kv1 displacement are present in the NAWM of MS brains at regions remote from lesions and are accompanied by axonal changes, which could be reproduced in a rat model of chronic meningeal inflammation that leads to chronic microglial activation throughout the brain. Paranodal disruption correlated with the presence of activated microglia, suggesting a mechanism of axonal injury that starts at the paranode independently of the demyelination process. Furthermore, we demonstrate that similar PNJ pathology could be induced in ex vivo cerebellar slice cultures by the activation of microglia with IFNγ and TNF or LTα and resultant glutamate release. Finally, we used biophysical simulations to systematically explore the effects on conduction of a range of paranodal and juxtaparanodal structural alterations observed in the human tissue and animal model.

## Results

### The PNJ structure is disrupted in postmortem MS NAWM

**Fig 1:**
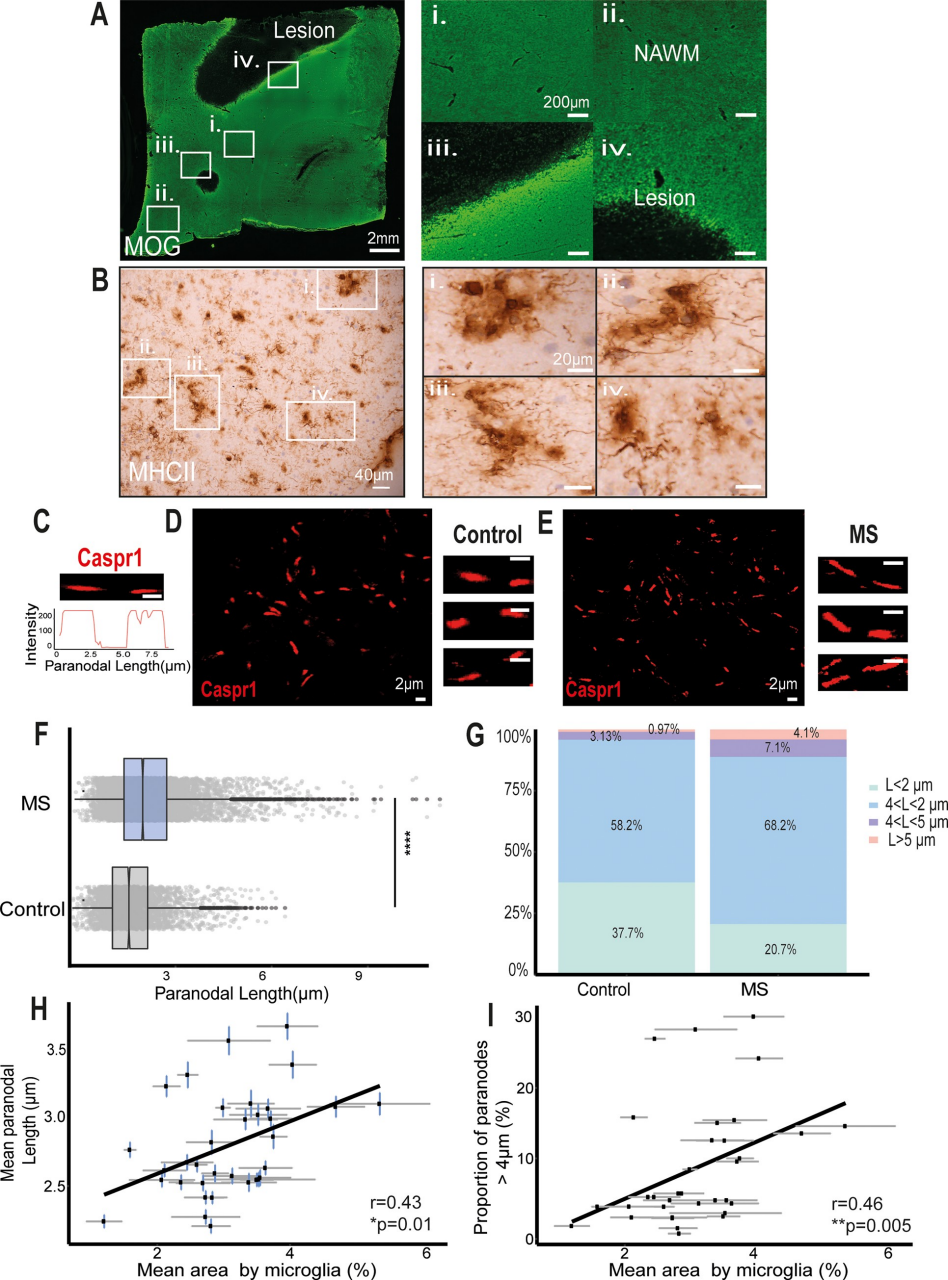
MS NAWM regions contained a larger proportion of elongated paranodes associated with activated microglia. (A) Anti-MOG-myelin immunofluorescence was used to identify areas of demyelination and normal appearing myelin. NAWM regions of interest (i, ii) were selected as areas distant from demyelinating lesions (iii, iv). (B) Clusters of process bearing anti-HLA-DR+ microglia with an activated morphology were found throughout the NAWM regions of interest and are illustrated at higher magnification in panels i–iv. (C) Confocal image of single Caspr1-stained paranode in cross-section and its intensity profile. (D, E) Confocal images of paranodes from human postmortem non-neurological control (D) and NAWM MS tissue (E). (F) Significantly different distributions of Caspr1+ paranodal lengths occurred in NAWM MS tissue in comparison to non-neurological control tissue (*p* < 0.0001, Mann–Whitney test). (G) NAWM MS tissue contained a larger proportion of Caspr1+ paranodes longer than 4 μm and 5 μm than the control tissue. (H) The mean paranodal length per block correlated with the mean area occupied by HLA-DR+ microglia/ macrophages (r = 0.43, *p < 0.5, Spearman rank correlation test). (I) The mean area occupied by HLA-DR+ microglia/macrophages per block correlated with the proportion of paranodes longer than 4 μm (r = 0.46, **p < 0.01, Spearman rank correlation test). MOG, myelin oligodendrocyte glycoprotein; MS, multiple sclerosis; NAWM, normal-appearing white matter. Data and code to reproduce this figure can be found at: https://github.com/PatGal2020/PLOS_submission

In order to characterise PNJ pathology present in human MS NAWM tissue and its relationship to local microglial activation and axonal cytoskeleton disruption, NAWM regions of interest at least 4 to 5 mm away from a demyelinating lesion were carefully selected from snap-frozen tissue blocks incorporating the cerebral peduncle and the precentral gyrus, both of which have a high density of longitudinal axons. Immunofluorescence with antibodies to the myelin protein, myelin oligodendrocyte glycoprotein (MOG), confirmed the myelin integrity (Fig 1A), and HLA-DR (MHC class II) antibodies confirmed the presence of microglia with an activated morphology (thicker and shorter processes) (Fig 1B). Immunofluorescence for Caspr1 localised at the paranodes demonstrated that Caspr1-stained paranodes (2.8 ± 1.1 μm) were significantly 21.7% longer on average in the MS NAWM tissue than in the non-neurological control tissue (2.3 ± 0.8μm, Fig 1C–1G). Furthermore, 43% of the paranodes in the MS tissue were longer than the 75% percentile of the control group (2.7 μm), and 11.14% were longer than 4 μm, compared to the 4.1% in the non-neurological controls (Fig 1G). There was little difference between the 2 brain areas examined; the paranodal lengths in the cerebral peduncles of the mesencephalon were 2.81 ± 0.019 μm long, and 12% of them were >4 μm, compared to those in the precentral gyrus, which were 2.79 ± 0.017 μm long, and 10% of them were >4 μm (S1 Fig). This suggests that the length of the paranodes, defined by Caspr1 labelling, was disrupted in a significant proportion of axons in MS brains compared to non-neurological controls.

### PNJ disruption is associated with microglial activation and axon stress

**Fig 2:**
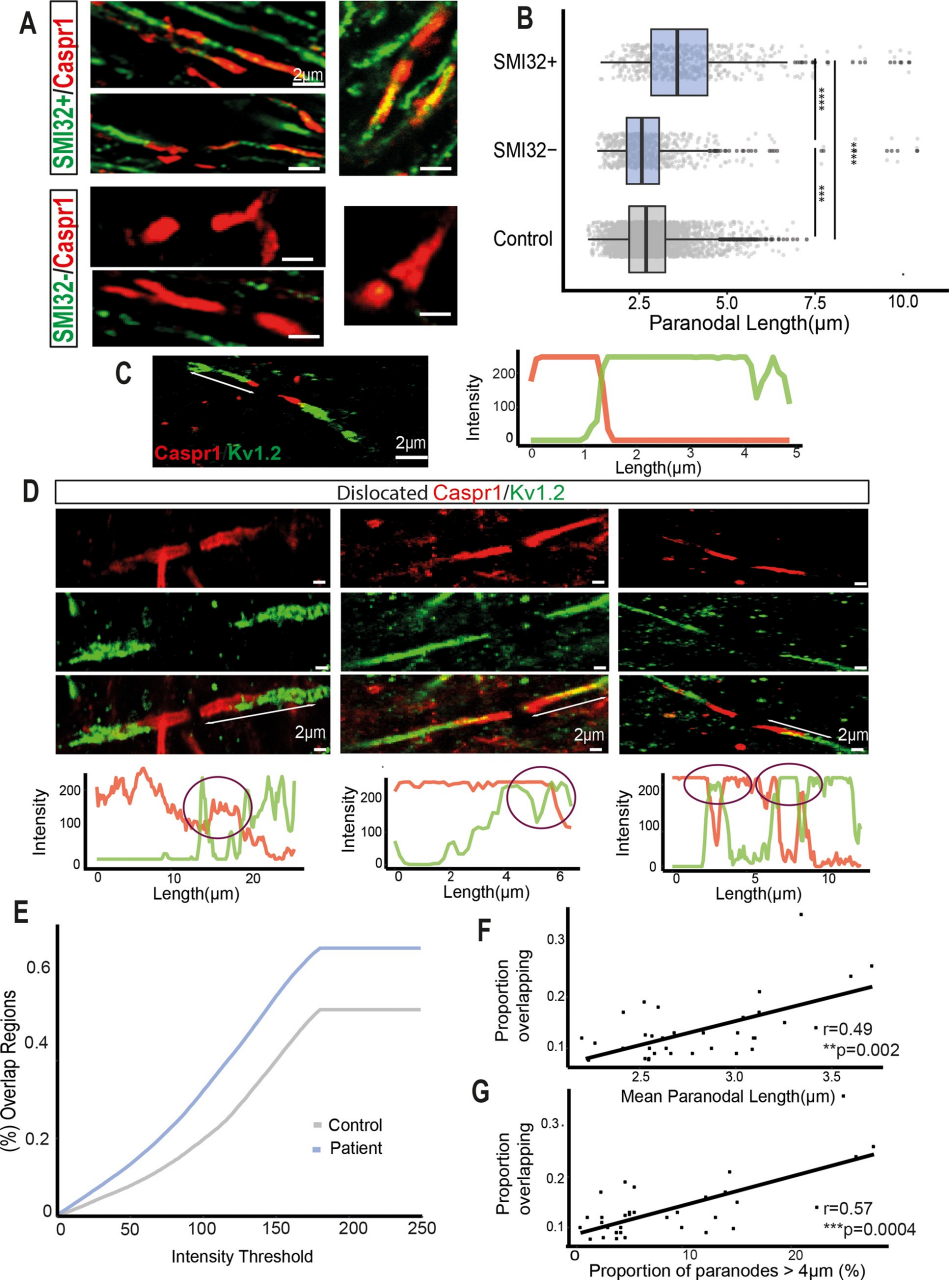
Paranodal elongation was associated with SMI32+ axons and the dislocation of juxtaparanodal voltage-gated Kv1.2 channels. (A) Confocal images of long Caspr1+ paranodes co-stained with SMI32 antibody. SMI32+ axons characterised by dephosphorylated neurofilaments had elongated paranodes. (B) Paranodal length distributions of SMI32+ and SMI32− axons from MS NAWM and non-neurological control tissue (**** *p* < 0.0001, Mann–Whitney test). (C) Confocal image from a node showing the expression of Caspr1 in the paranode (red) and K_v_1.2 channels in the juxtaparanodes (green) do not overlap under non-pathological conditions and the respective RGB profile. (D) Confocal images from nodes where Caspr1 and K_v_1.2 are colocalising, and therefore possibly being affected by MS neuropathological conditions and their intensity RGB profiles. The purple circles denote regions where both signals are colocalising: overlapping regions. (E) When the difference between Caspr1 and K_v_1.2 signals was smaller than a variable intensity threshold, we considered that point as an overlapping region. For every threshold calculated, the proportion of overlapping regions was larger in MS NAWM tissue (blue) than in non-neurological control tissue (grey). (F) The mean paranodal length per block correlated with the proportion of overlapping regions at an intensity threshold of 50 (r = 0.49, **p < 0.01, Spearman rank correlation test). (G) The proportion of paranodes longer than 4 μm per block correlated with the proportion of overlapping regions at a threshold of 50 (r = 0.57, ***p < 0.001, Spearman rank correlation test). MS, multiple sclerosis; NAWM, normal-appearing white matter. Data and code to reproduce this figure can be found at: https://github.com/PatGal2020/PLOS_submission.

Chronic activation of microglia and axonal degeneration are two of the main pathological features of NAWM tissue in progressive MS. Therefore, we examined their relationship to paranodal length as a marker of paranodal axo-glial disruption. The mean area occupied by HLA-DR+-labelled microglia was obtained as a measurement of microglial activation. Moderate significant correlations were found between mean paranodal length and the proportion of paranodes >4 μm and the mean microglial area (r = 0.46 and r = 0.43, *p < 0.05, Fig 1H and 1I). Double immunofluorescence labelling with the SMI32 antibody, which labels dephosphorylated neurofilament proteins, an indicator of axon stress, and Caspr1 to indicate paranodal length (Fig 2A), demonstrated that SMI32+ axons had longer Caspr1-stained paranodes on average (mean = 3.89 ± 0.1 μm) than SMI32− (mean = 2.49 ± 0.07 μm, Fig 2B) and non-neurological controls (mean = 2.33 ± 0.01 μm). Furthermore, the average paranodal length for SMI32+ axons was 66.9% longer than the control paranodal length and 56.2% longer than SMI32− axon paranodal length (Fig 2B), indicating a strong relationship between the altered paranodes and axonal stress.

### Paranodal disruption is associated with juxtaparanodal K_v_1.2 channel dislocation

One of the roles of the tightly adherent axo-glial junctions is to promote the clustering and segregation of voltage-gated channels and prevent electrical current shunting underneath the myelin sheath. To examine if paranodal structural instability could provoke a dislocation of juxtaparanodal voltage-gated K_v_1.2 channels to the paranode in MS NAWM tissue, RGB intensity profiles of the Caspr1 (red) and voltage-gated K_v_1.2 channel (green) labelled paranodes and juxtaparanodes were measured (Fig 2C and 2D). The colocalisation of Caspr1 and K_v_1.2 was calculated by subtracting both intensity signals one from each other. When this difference was smaller than a variable threshold, we took that point as an overlapping region (source code in S1 Text). For a variable intensity threshold, MS NAWM tissue had a higher proportion of overlapping regions at every threshold than the non-neurological control tissue (Fig 2E). For example, if the threshold was set to 50, the MS NAWM tissue had 19.7% more overlapping regions than non-neurological control tissue, while at a threshold of a 100, the MS NAWM tissue had 41% more overlapping regions than control. Thus, paranodal disruption was accompanied by a dislocation of the juxtaparanodal K_v_1.2 channels towards the node, making these voltage-gated channels more exposed to the more extensive nodal extracellular space, which is more capacitive and less resistive than the compacted myelin of the juxtaparanodes. Additionally, significant correlations were present between the proportion of overlapping regions in NAWM tissue when the threshold was set to 50 and the mean paranodal length (Fig 2F) and proportion of paranodes longer than 4 μm (Fig 2G). The same quantification procedure was followed for assessing dislocation of the nodal Na_v_ voltage-gated channels into the PNJ, but no significant dislocation was identified, even in the presence of elongated paranodes (S2 Fig). Overlap between Na_v_ voltage-gated channels and components of the paranodal junctions was not observed.

### Prolonged exposure of the rat cortex to pro-inflammatory cytokines can generate paranodal disruption

**Fig 3:**
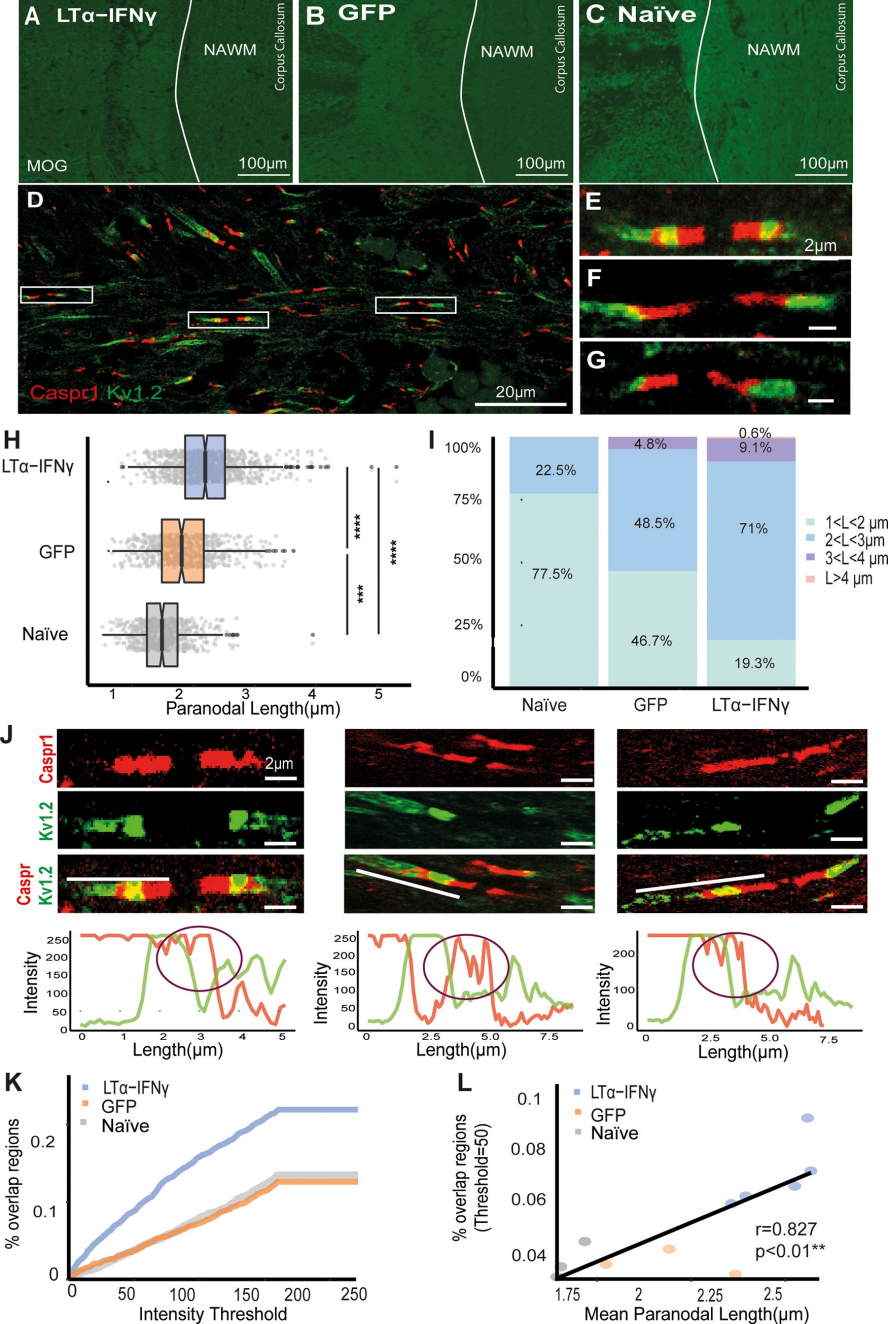
LTα/IFNγ-affected rat tissue contained a higher proportion of elongated Caspr1-paranodes compared to the GFP and naive. (A–C) Immunofluorescent images of MOG-stained corpus callosum, cingulate, and external capsule of the LTα/IFNγ, GFP, and naive rats. (D) Confocal image of Caspr1-K_v_1.2-stained paranodes and juxtaparanodes from a LTα/IFNγ rat. (E–G) Confocal images of single Caspr1-K_v_1.2-stained paranodes and juxtaparanodes from a LTα/IFNγ rat. (H) Caspr1-measured paranodal length distributions of LTα/IFNγ (blue), GFP (orange), and naive (grey) rat groups (**** *p* < 0.0001, Mann–Whitney test). (I) Paranodal length data were divided into different length ranges and represented in a bar plot. LTα/IFNγ vector-injected brains contained a larger proportion of paranodes longer than 3 and 4 μm. (J) Confocal images in which Caspr1-stained paranodes colocalised with K_v_1.2-stained juxtaparanodes. In order to quantify the displacement of the channels, the RGB intensity profiles of each paranode and juxtaparanode were acquired. The purple circles denote regions where both signals were colocalising. (K) Graph showing that the proportion of overlapping regions between Caspr1 and K_v_1.2 RGB signals was larger at every intensity threshold in the LTα/IFNγ group compared to the GFP and naive groups. (L) Mean paranodal length correlated significantly with the proportion of overlapping regions when the intensity threshold was set to 50 (r = 0.827, **p < 0.01, Spearman rank correlation test). GFP, green fluorescent protein; IFNγ, interferon-γ; LTα, lymphotoxin-α; MOG, myelin oligodendrocyte glycoprotein. Data and code to reproduce this figure can be found at: https://github.com/PatGal2020/PLOS_submission.

To further study the relationship between chronic inflammation in the NAWM and PNJ pathology, we used a novel rat model of chronic meningeal inflammation [38]. In this model, inflammation was initiated by chronic production of the pro-inflammatory cytokines LTα and IFNγ within the meninges and cerebrospinal fluid (CSF). Meningeal inflammation induced widespread microglial activation over 3 months in the absence of WM demyelination, which did not occur in these animals (S3 Fig). This allowed the study of the effects of diffuse neuroinflammation on the axons of the NAWM, similar to those seen in progressive MS, avoiding Wallerian degeneration and dying back axonal injury patterns resulting from demyelination. Control groups were rats injected with a green fluorescent protein (GFP) gene vector or naive rats (S3 Fig). NAWM regions were selected from the MOG immunofluorescence images of the corpus callosum, cingulate, and external capsule (Fig 3A–3C).

Immunofluorescence analysis of Caspr1 and voltage-gated K_v_1.2 channel localisation was carried out as for the human tissue (Fig 3D–3G). In total, 1,000 Caspr1-stained paranodes (200 paranodes per rat) from the LTα/IFNγ group, 600 from the GFP group, and 600 from the naïve group were analysed. The mean Caspr1-stained paranodal length in the LTα/IFNγ vector group (2.41 ± 0.01 μm) was 35.6% longer than values for naive rats (1.77 ± 0.01 μm) and 15.9% longer than the GFP-vector-injected rats (2.08 ± 0.02 μm). Furthermore, 82% of the paranodes in the LTα/IFNγ group were longer than the 75% percentile of the naïve group (1.98 μm) (Fig 3H). Paranodal length distributions of each group showed that 9.7% of the paranodes in the LTα/IFNγ group were longer than 3 μm compared to the 4.8% in the GFP group and 0% in the naïve group (Fig 3I (purple and pink)). Compared to the naïve group, in which 77.5% of the paranodes were shorter than 1 μm, in the GFP group 46.7% and in the LTα/IFNγ group 19.3% were shorter that 1 μm in length (Fig 3I). Thus, paranodal axo-glial junctions in the LTα/IFNγ vector-injected animals, in which there was widespread microglial activation, were highly disrupted compared to the GFP and naïve groups. Interestingly, the GFP vector injected animals also had a modest but significant increase in paranodal length, along with a low level of microglial activation, but at a level that was significantly less than the cytokine vector-injected animals.

Analysis of the RGB intensity profiles of Caspr1 and K_v_1.2 channels, following the same method as in the postmortem human tissue (source code in S1 Text), showed that the brains of LTα/IFNγ vector-injected animals contained a larger proportion of axons with overlapping regions (Fig 3J and 3K) at every threshold analysed. Therefore, the LTα/IFNγ animals had more damaged axons with dislocated voltage-gated K_v_1.2 channels, indicating more disrupted PNJs than the GFP and naïve groups (Fig 3J and 3K). When the intensity threshold was set to 50, the proportion of overlapping regions were significantly different between LTα/IFNγ and GFP or naïve groups. Moreover, the proportion of overlap correlated with the average paranodal length (Fig 3L, r = 0.827) and with the proportion of paranodes >3 μm per rat (r = 0.765). These results suggest that paranodal lengthening and K_v_1.2 dislocation were often observed together. Although there was clearly an increase in the number of axons expressing SMI-32 immunoreactivity in animals injected with LTα/IFNγ vector (S3D Fig), similar to the MS NAWM, it proved impossible to quantify the relationship between these SMI32+ axons and Caspr1+ paranodal length due to the compact axon density in the corpus callosum, cingulate, and external capsule in the rat.

**Fig 4:**
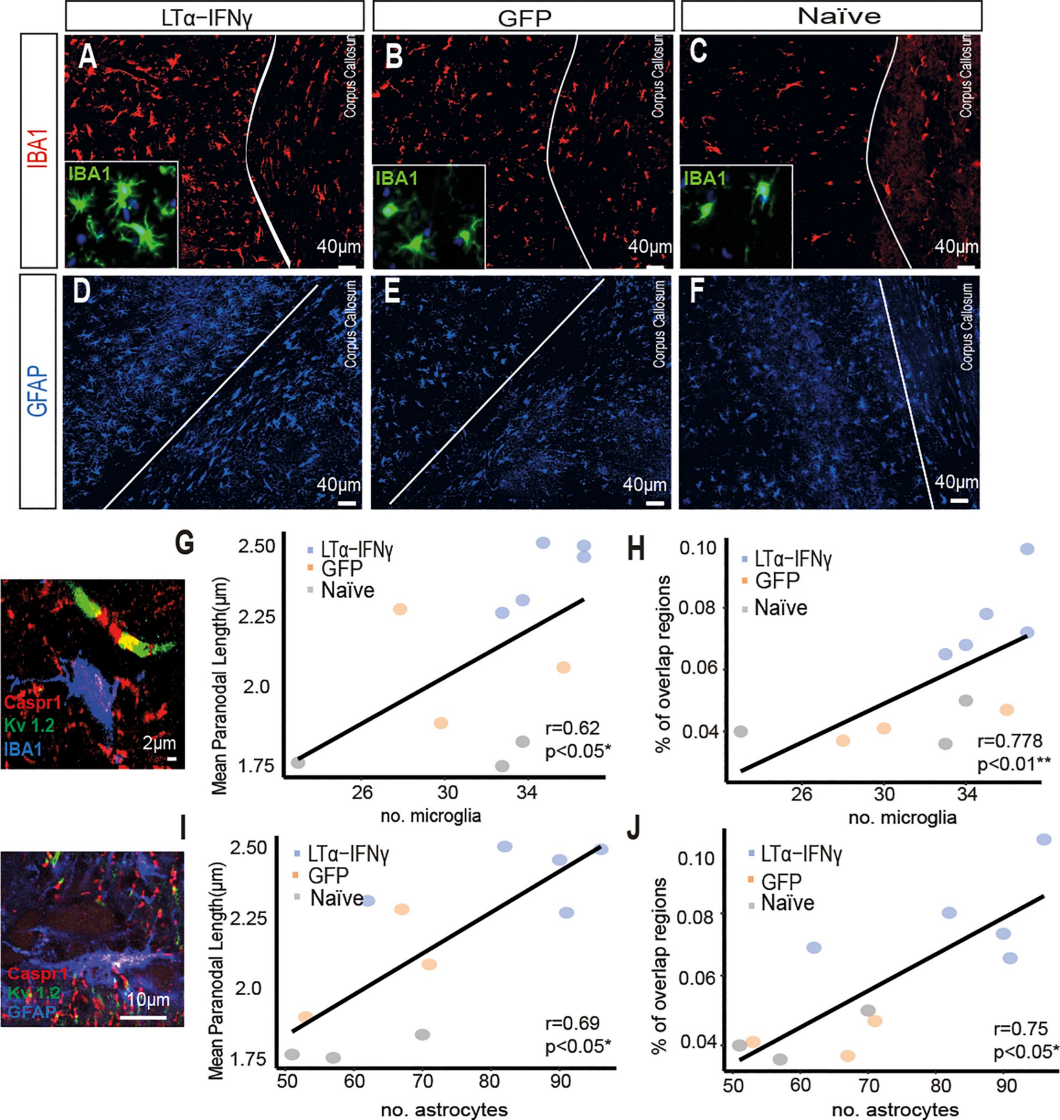
Paranodal lengthening and Kv1.2 dislocation occurs simultaneously, and they are linked to glial activation. (A–C) Immunofluorescent images of IBA1+ microglia (red) from the corpus callosum of LTα-IFNγ, GFP, and naive rats. Insets show that microglia (shown in green) display a highly activated morphology in the cytokine vector-injected animals compared to the GFP control and naive animals. (D–F) Immunofluorescent images of GFAP+ astroglia from the corpus callosum of the LTα/IFNγ, GFP, and naive rats. (G) The number of microglia correlated with the mean paranodal length per rat (r = 0.62, *p < 0.05, Spearman rank correlation test). (H) The number of microglia correlated with the proportion of overlapping regions between Caspr1 and K_v_1.2 when the intensity threshold was set to 50 (r = 0.778, **p < 0.01, Spearman rank correlation test). (I) The number of astroglia correlated with the mean paranodal length per rat (r = 0.69, *p < 0.05, Spearman rank correlation test). (J) The number of astroglia correlated with the proportion of overlapping regions between Caspr1 and K_v_1.2 when the intensity threshold was set to 50 (r = 0.75, *p < 0.05, Spearman rank correlation test). GFP, green fluorescent protein; IFNγ, interferon-γ; LTα, lymphotoxin-α. Data and code to reproduce this figure can be found at: https://github.com/PatGal2020/PLOS_submission.

To investigate whether surrounding inflammation could have a role in this pathology, the number of microglia and astroglia in the NAWN regions were assessed on serial sections from the same animals (Fig 4A–4F). The number of microglia in the NAWM correlated significantly with the average paranodal length (Fig 4G, r = 0.62) and with the proportion of overlapping regions between Caspr1 and K_v_1.2 (Fig 4H, r = 0.778). Although there was no significant difference in total microglial numbers between the groups, microglia in the LTα/IFNγ vector-injected animals had an activated morphology compared to the GFP vector-injected and naive animals (insets in Fig 4A–4C). Furthermore, the number of astroglia also correlated with the average paranodal length (Fig 4I, r = 0.69) and with the proportion of overlapping regions between Caspr1 and K_v_1.2 (Fig 4J, r = 0.75). These data support our hypothesis that points to microglia and astroglia as major mediators of the paranodal axo-glial junction disruption in myelinated axons.

### TNF/IFNγ-activated microglia and astroglia release high levels of glutamate

**Fig 5:**
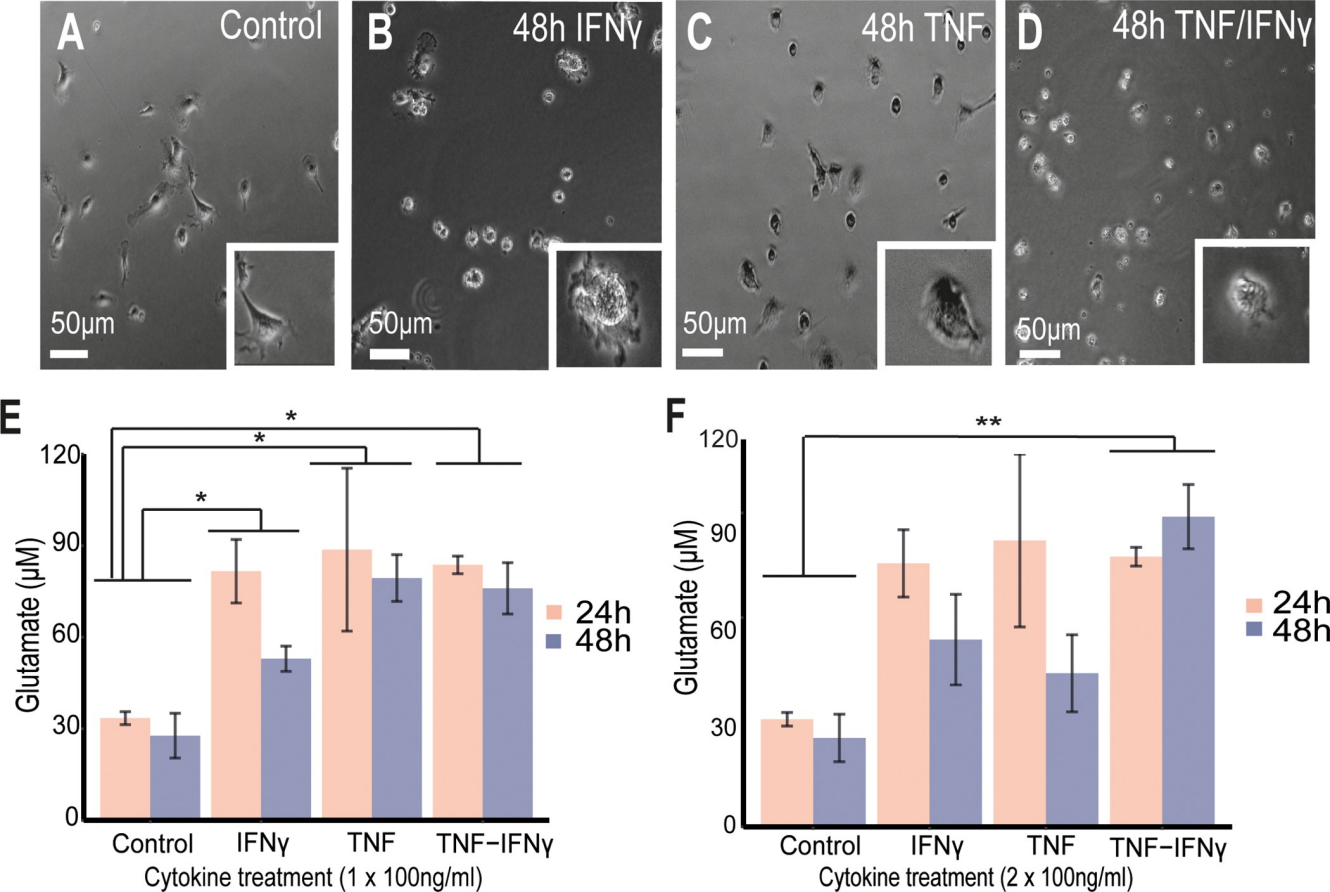
TNF/IFNγ-activated microglia release high amounts of glutamate. (A) Live image of nontreated primary microglial cultures, (B) cultures treated with IFNγ after 48 h, (C) cultures treated with TNF after 48 h, and (D) cultures treated with TNF/IFNγ after 48 h. (E, F) Mean ± SEM for glutamate levels from replicates showing the statistical difference between controls and the cytokine treatments: (E) 100 ng/ml (*n* = 3 Control, *n* = 3 TNF, *n* = 3 IFNγ, *n* = 3 TNF/IFNγ), (F) 2 acute treatments of 100 ng/ml (*n* = 3 Control, *n* = 3 TNF, *n* = 3 IFNγ, *n* = 4 TNF/IFNγ). Nonparametric Friedman test was performed across cytokine groups and timings and post hoc paired-wised Wilcoxon tests to compare groups (* *p* < 0.05, ** *p* < 0.01). IFNγ, interferon-γ; TNF, tumour necrosis factor. Data and code to reproduce this figure can be found at: https://github.com/PatGal2020/PLOS_submission.

Previous studies have suggested that elevated glutamate levels are able to induce nodal changes [17–19]. Therefore, in order to assess if increased levels of pro-inflammatory cytokines in the CNS can stimulate glutamate release from microglia and/or astroglia, primary rat microglial and astroglial cultures were treated with different concentrations of TNF, IFNγ, or TNF + IFNγ (S4A Fig). Microglia were activated by TNF and/or IFNγ as indicated by transformation of their morphology from a ramified shape to a more activated amoeboid morphology (Fig 5A–5D). A single treatment with 100 ng/ml of TNF, IFNγ, or TNF+IFNγ, induced a significant increase in glutamate release after 24 h (TNF: 88.68 ± 26.67 μM, IFNγ: 81.62 ± 10.4 μM, TNF + IFNγ: 83.69 ± 2.88 μM) and 48 h (TNF: 79.39 ± 7.64 μM, IFNγ: 52.95 ± 4.14 μM, TNF + IFNγ: 76.02 ± 8.42 μM) compared to controls (24 h: 33.49 ± 2.11 μM; 48 h: 27.73 ± 7.33 μM) (Fig 5E), which did not increase further if the dose was increased to 200 ng/ml (S4B Fig). The difference in glutamate levels between 24 h and 48 h across treatments was not significant (Fig 5E). However, administration of 2 doses of 100 ng/ml of TNF + IFNγ 24 h apart (Fig 5F) resulted in the highest concentration of glutamate in the medium at 48 h (96.05 ± 9.92 μM) when compared to the single treatments. We have used TNF and LTα interchangeably here as they both act via the TNFR1 receptor in this context. The same experiments on the primary microglial cultures were also carried out with the pro-inflammatory cytokines LTα and IFNγ, which also resulted in a significant glutamate release compared to controls after 2 doses administered 24 h apart (S4C and S4D Fig). In a similar way, TNF + IFNγ also stimulated significant glutamate release from astroglia 24 h after addition at both 100 ng/ml and 200 ng/ml (S5A Fig), although there was no significant difference between 100 and 200 ng/ml. In addition, glutamate uptake by astroglia was substantially inhibited by the addition of the cytokines (S5B Fig).

### TNF/IFNγ and direct glutamate administration induce paranodal elongation in ex vivo cerebellar slices

**Fig 6:**
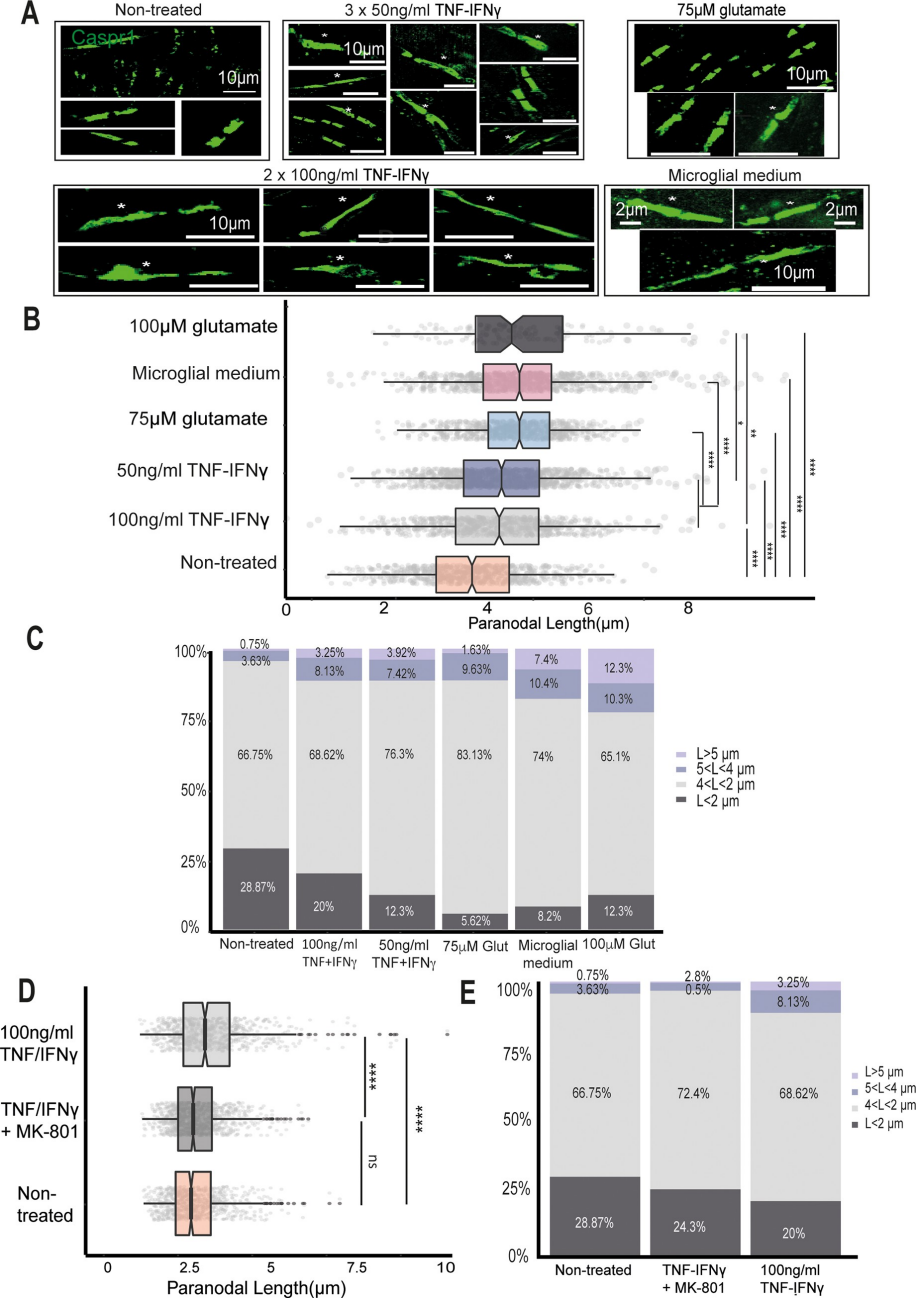
The proportion of elongated paranodes in cerebellar tissue slices treated with pro-inflammatory cytokines and glutamate. (A) Confocal images of Caspr1-stained paranodes from nontreated cerebellar cultures, slices treated with 3 doses of 50 ng/ml TNF/IFNγ, slices treated with 2 doses of 100 ng/ml TNF/IFNγ, slices treated with 2 doses of 75 μM glutamate, and slices treated with 2 doses of microglial-conditioned medium (medium from primary microglia treated with 2 doses of 100 ng/ml TNF/IFNγ); asterisks point to long and disrupted paranodes. (B) Box-plots showing the different paranodal length distributions between the treated and nontreated cultures (nonparametric Kruskal–Wallis test and post hoc Wilcoxon rank sum test, ****p < 0.0001). (C) Bar plots of the same paranodal length distributions showing the proportion of paranodes in each data set of different lengths. (D) Box-plot of the paranodal length distributions of the nontreated cultures (orange), 2 doses of 100 ng/ml TNF + IFNγ with MK-801 (dark grey), and 2 doses of 100 ng/ml TNF + IFNγ alone (light grey) (**** *p* < 0.0001, Mann–Whitney test). (E) Bar plot showing the same paranodal length distributions divided into different length ranges. IFNγ, interferon-γ; TNF, tumour necrosis factor. Data and code to reproduce this figure can be found at: https://github.com/PatGal2020/PLOS_submission.

In order to determine if TNF + IFNγ and glutamate could cause MS-like paranodal pathology, organotypic cerebellar slices derived from P8/9 rats and cultured for 8 to 10 days were treated with 3 doses of 50 ng/ml TNF/IFNγ, 2 doses of 100 ng/ml of TNF/IFNγ, 2 doses of microglial-conditioned medium, 2 doses of glutamate at 75 μM, or 2 doses of glutamate at 100 μM (in all the treatments, doses were administered every 24 h, and glutamate levels were measured 24 h after the last dose (S6 Fig). For each of the cerebellar tissue slices, 200 focused Caspr1-stained paranodes were analysed (Fig 6A). In cerebellar slices treated with TNF/IFNγ, Caspr1-stained paranodal length was 19.15% longer on average in the 50 ng/ml group (2.96 ± 0.03 μm) and 16.6% on average in the 100 ng/ml group (2.88 ± 0.04 μm) than in the nontreated cerebellar cultures (2.47 ± 0.03 μm, Fig 6B). However, when separating the paranodal measurements by length ranges, they had larger proportions of highly disrupted long paranodes >4 μm than the untreated cerebellar slices (11.38% in the 100 ng/ml group and 11.34% in the 50 ng/ml group; Fig 6C, light and dark purple). Abnormally long Caspr1-stained paranodes were also observed (asterisks in Fig 6A).

Five cerebellar slices were treated with the primary microglial-conditioned medium produced after 2 doses of 100 ng/ml of TNF/IFNγ, the condition that resulted in the greatest glutamate release. These cerebellar cultures had an average paranodal length of 3.23 ± 0.04 μm, 32.84% longer on average than the nontreated ones (2.47 ± 0.027 μm; 200 paranodes were analysed per cerebellar slice, Fig 6A–6C). In order to examine if glutamate was one of the main cytotoxic factors in the microglial cytokine-conditioned medium, 4 cerebellar slices were treated with 75 μM of glutamate for 48 h (the average glutamate concentration in the 100 ng/ml TNF/IFNγ group was 96.046 ± 9.92 μM and the conditioned medium was diluted 4:1), which resulted in paranodes 26.11% longer on average than the nontreated tissue slices (Fig 6A–6C). In order to confirm if the effect of TNF/IFNγ was due to glutamate release and action, 5 slices were treated with 2 doses of 100 ng/ml of TNF/IFNγ together with the noncompetitive NMDA antagonist MK-801 (0.6mM) (Fig 6D). The paranodal length distribution of the slices was not significantly different between the MK-801-treated and nontreated slices (Fig 6E). The proportion of paranodes longer than 4 μm was greatly reduced in the MK-801 group compared to the cultures treated with the cytokines alone (from 11.38% of the paranodes to 3.3%, Fig 6E).

### PNJ disruption can affect velocity and conduction in small-diameter axons

**Fig 7:**
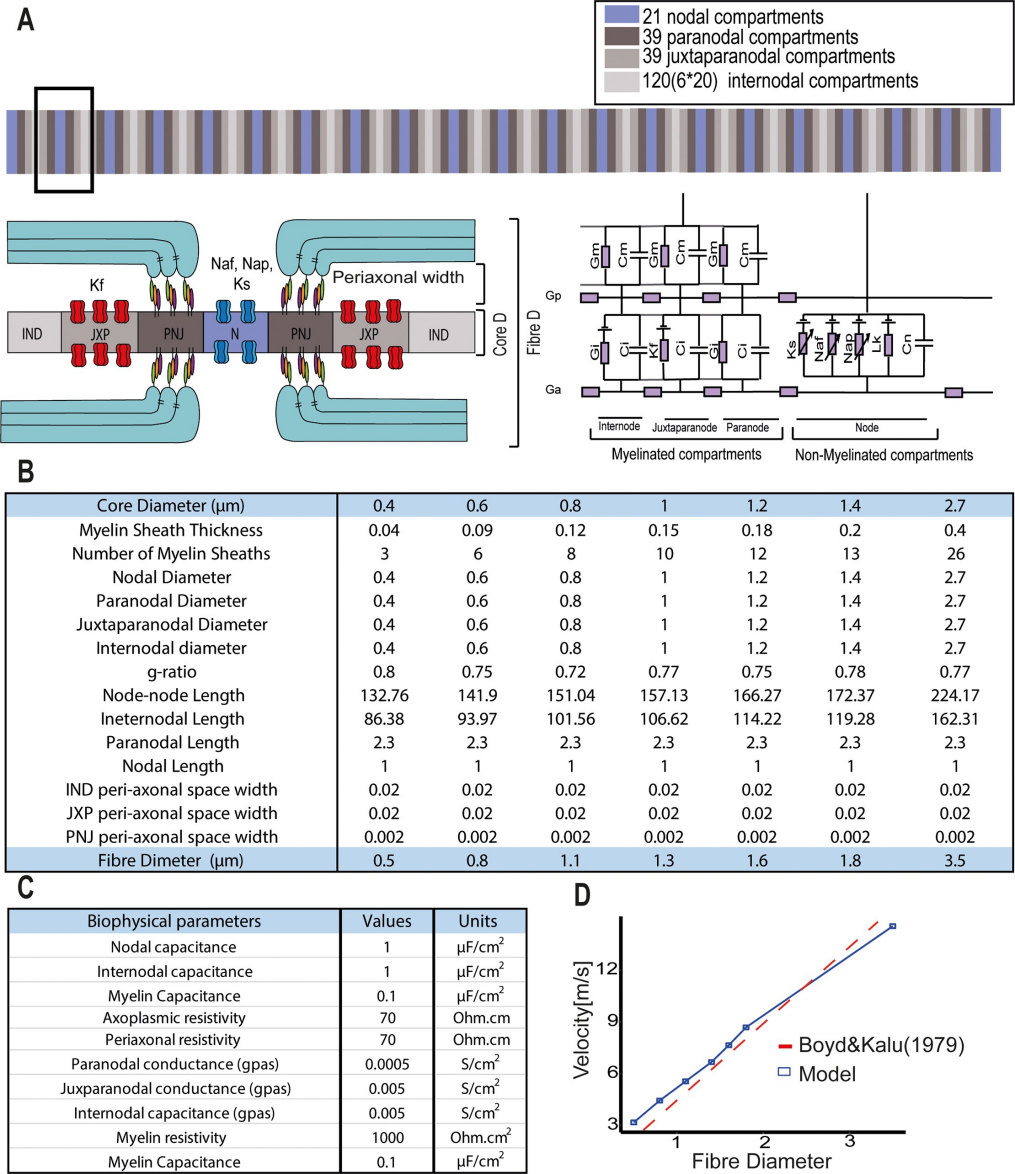
Structural and biophysical parameters used to simulate a 21 node myelinated axon. (A) A double cable circuit of the model to represent the axolemma (Ga), the peri-axonal space (Gp), and the myelin sheath (Gm) was generated with the simulator NEURON. Specifically, 21 nodal, 39 paranodal, 39 juxtaparanodal, and 20 internodal compartments were created. (B) Anatomical parameters used in the model. Seven diameters were chosen from CNS measurements from macaque EM studies [42], the number of myelin lamella (nl) was calculated from the myelin periodicity value of 0.0156 μm [71], the node-to-node length was taken from the linear relationship measured from rat nerve fibres [72], the juxtaparanodal length was extrapolated from the diameter-dependent scaling relationship from the ventral root of cats [73], and the paranodal length was determined from the average value of Caspr1 staining measured from our non-neurological control cases. (C) Biophysical parameters. Axon capacitance was based on data from rat ventral roots [86], myelin capacitance and leak conductance per lamella were based on the frog sciatic nerves [87]. The resistivity was set to 1,000 Ohm * cm^2^ for each myelin lamella [40,87,88,89,90,91]. (D) Plot showing the conduction velocity of the model across the 7 fibre diameters simulated (blue) and the velocity data measured in cat hind limb nerves [43].

To systematically examine the consequences of paranodal disruption on AP propagation through the use of computational modelling, a double cable core model was built and solved numerically in NEURON (Fig 7A). A double cable core-conductor circuitry was chosen to represent the biophysical parameters of the axonal membrane, the peri-axonal space, and the myelin sheath separately [39–41]. We simulated the functionality of an axon membrane made up of 4 types of compartments: nodes, paranodes, juxtaparanodes, and internodes. The nodes clustered a high density of fast Na_v_ channels (Na_v_1.6), persistent Na_v_ channels, and slow K_v_ channels (Fig 7A, purple). Immediately flanking the nodes, the paranodes were built as compartments with no active conductances, and the sites where the myelin end loops connect with the axolemma (Fig 7A, dark grey). Next to the paranodes, the juxtaparanodes contained fast K_v_ channels (Fig 7A, medium grey). Finally, the internodes were sections surrounded by myelin with a low density of ion channels (Fig 7A, light grey). Seven fibre diameters were simulated: d _fibre_ = 0.5, 0.8, 1.1, 1.3, 1.6, 1.8, 3.5 [μm]. These small-caliber diameters were chosen taking into consideration previous human brain and macaque electron microscopy (EM) studies, which indicated that the average axon core diameter within the CNS is 1 μm [42]. All the structural and biophysical parameters used in the simulations were taken from previous EM studies and are summarised in Fig 7B and 7C, whereas the conductances and gating dynamics of the channels were based on previous electrophysiological studies, detailed in the Materials and methods. The conduction velocity predicted by our simulations showed a linear relationship with the fibre diameter (V[m/s] = 4.52 * d_fibre_ [μm]) (Fig 7D, blue line), which reproduced previous experimental results (Fig 7D, dotted grey line, velocity data derived by Boyd and Kalu [43] from small diameter axons in the cat hind limb nerves V = 4.6 * d_fibre_).

**Fig 8:**
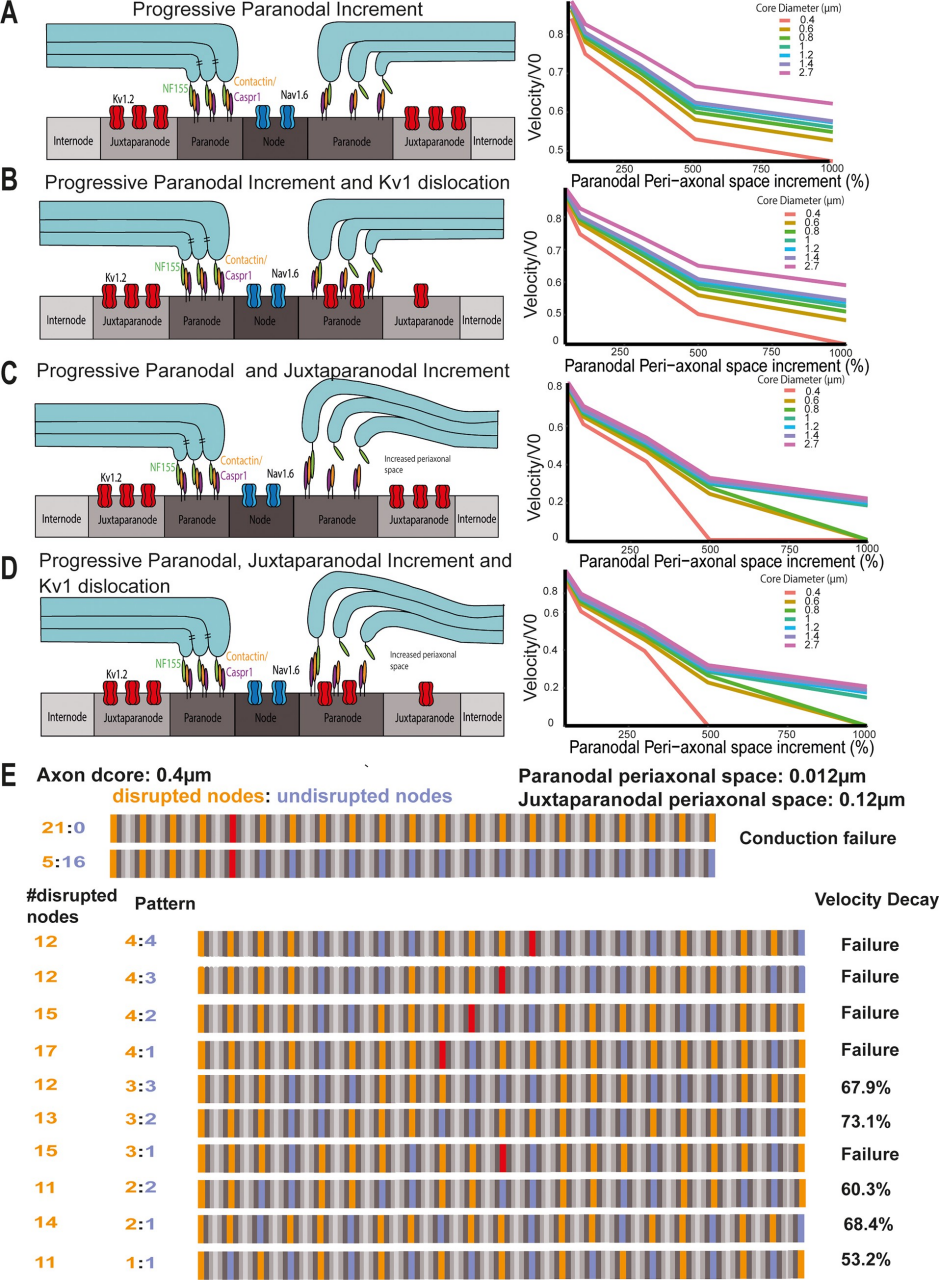
The effects of paranodal disruption on AP conduction is inversely proportional to the axon diameter. (A) Diagram representing the increment of the paranodal peri-axonal space and a normalised velocity plot at every core diameter as the paranodal peri-axonal space increases. (B) Diagram representing the increment of the paranodal and juxtaparanodal peri-axonal space and a normalised velocity plot at every core diameter as the paranodal peri-axonal space increases. (C) Diagram representing the increment of the paranodal and juxtaparanodal peri-axonal and K_v_1 dislocation and a normalised velocity plot at every core diameter as the paranodal peri-axonal space increases. (D) Diagram representing the increment of the paranodal and juxtaparanodal peri-axonal and K_v_1 dislocation and a normalised velocity plot at every core diameter as the paranodal peri-axonal space increases. (E) In an axon model with a core diameter of 0.4 μm, conduction failure occurred when 5 consecutive nodes were disrupted (orange), and the paranodal and juxtaparanodal peri-axonal space widths were increased up to 0.012 and 0.12 μm, respectively. Further, the velocity can decay and conduction can fail under different patterns of disruption (orange means disrupted node, purple, healthy node, and red denotes conduction failure).

In order to simulate paranodal disruption, the resistance of the paranodal and/or juxtaparanodal compartments was decreased, and the juxtaparanodal K_v_ channels were dislocated. The resistance was decreased by increasing the peri-axonal space of both compartments progressively. The observed paranodal lengthening was represented in this model by an increment in peri-axonal space on the assumption that if some myelin-end loops at the PNJ detached from the axolemma, these spaces will be progressively larger [20,21]. Furthermore, juxtaparanodal K_v_ channels were dislocated to the paranode, and their conductance was increased proportionally to the increment in peri-axonal space of the paranode. In summary, the following structural arrangements were simulated: (a) proportional increment of the paranodal peri-axonal space (Fig 8A); (b) proportional increment of the paranodal and juxtaparanodal peri-axonal space (Fig 8B); (c) proportional increment of the juxtaparanodal fast K_v_ channels conductance and the paranodal peri-axonal space (Fig 8C); and the proportional increment of the juxtaparanodal fast K_v_ channels conductance, and the paranodal and juxtaparanodal peri-axonal spaces (Fig 8D).

The conduction velocity decreased as the paranodal peri-axonal space increased for all the axon diameters (Fig 8A). Fitting the data to a logarithmic curve, the velocity of the axons decreased faster in the smaller-diameter axons than the larger-diameter ones as the paranodal peri-axonal space was increased (Fig 8A). When the juxtaparanodal fast K_v_ channels were dislocated to the paranodal compartment (Fig 8B), the velocity also decreased faster in the small-diameter axons, although the dislocation of the channels did not significantly alter this. When a progressive increment of the paranodal and juxtaparanodal peri-axonal spaces was introduced (Fig 8C), the axon with a core diameter of 0.4 μm failed to conduct when the paranodal and juxtaparanodal peri-axonal spaces were increased by 500% (the paranodal peri-axonal space was 0.012 μm and the juxtaparanodal 0.12 μm). Meanwhile, the axons with d_core_ of 0.6 μm and 0.8 μm also failed to conduct when the paranodal and juxtaparanodal peri-axonal spaces were increased by 1,000% (the paranodal peri-axonal space was 0.022 μm and the juxtaparanodal 0.22 μm). Additionally, larger-diameter axons had a significant velocity decay in the same conditions. For example, the velocity of the axon with a core diameter of 2.7 μm decreased 78.47% (y = −0.209 * ln(x) + 1.6457, r^2^ = 0.99). In the last structural arrangement, the paranode and juxtaparanode peri-axonal spaces were increased and the K_v_1 were dislocated (Fig 8D). In this condition, conduction failure occurred in the axons with a core diameter of 0.4, 0.6, and 0.8 μm and a decrease in axons with a core diameter of 2.7 μm. These data suggest that AP conduction in the smaller-diameter axons might be more susceptible to paranodal and juxtaparanodal disruption than larger-diameter axons.

In the previous 4 conditions, all 21 nodes of the axon were disrupted by increasing the peri-axonal space widths and displacing the juxtaparanodal voltage-gated K_v_ channels. However, conduction failure only occurred in the small-diameter axons (d_core_ of 0.4, 0.6, and 0.8 μm). Therefore, we also examined the number of consecutive nodes needed for conduction failure at these diameters, and the velocity decay after simulating conduction in an axon where patches of healthy nodes (Fig 8E, purple) were interspersed with patches of disrupted nodes (Fig 8E, orange), which is more likely to reflect the pathological situation. In the axon with d_core_ = 0.4 μm, 5 consecutive nodes were needed for conduction failure when the width of the paranodal and juxtaparanodal peri-axonal spaces were increased to 0.012 and 0.12 μm, respectively (Fig 8E). We then explored if damaging less than 5 nodes consecutively in different patterns would also cause conduction failure or a decrease in conduction velocity (Fig 8E). By simulating different patterns, we could observe that the total number of disrupted nodes along an axon did not determine if the conduction was going to fail, but it depended on the number of consecutive nodes disrupted. For example, when 3 disrupted nodes (Fig 8E, orange) were followed by 2 healthy nodes (Fig 8E, purple), the conduction did not fail, instead it caused a velocity decay of 73.1%. Meanwhile, in other conditions with less numbers of disrupted nodes, the conduction failed when 4 consecutive nodes were disrupted (Fig 8E). Simulations using different patterns for axons with d_core_ = 0.6 and 0.8 μm can be found in S7 and S8 Figs.

## Discussion

The nodes of Ranvier represent the only points of direct contact between myelin and the axon, specifically at the paranodal axo-glial junctions [14,44], and it is suggested that these sites could be one of the targets of the immune mediated attacks in MS. Paranodal and juxtaparanodal pathology has been described in only a few studies in human MS tissue [16,45–47], but its effects and causes remain unknown. Our detailed analysis of the longitudinal elongation of the Caspr1-stained paranodal axo-glial junctions and dislocation of the K_v_1.2 channels indicate a clear disruption in a proportion of paranodes in MS NAWM compared to non-neurological control tissue. This extends and supports our previous study that showed an elongation of the glial neurofascin 155 expressing paranodal structures [16]. Here we report significant alterations in caspr1+ paranode length, juxtaparanodal disruption, and the association with axon stress/damage, across a broad spectrum of NAWM samples, defined by the extent of microglial activation as a continuous variable. Previously, such an association was only demonstrated in binary groups of MS NAWM displaying “low” or “high” microglial activation. These findings were reproduced in both an in vivo and an ex vivo model by the presence of persistently elevated levels of TNF/LTα and IFNγ, which indicates that pro-inflammatory cytokines could be the trigger for this pathology. Further investigations using primary cultures and organotypic cerebellar slice cultures suggested that glutamate release by microglia and/or astroglia, in response to stimulation with pro-inflammatory cytokines, could mediate these pathological changes at the paranodes. Accumulation of abnormal nodes of Ranvier could be responsible for some of the generalised MS symptoms that cannot be attributed to focal lesions, such as the fatigue that is reported as a major symptom by 70% to 90% of MS patients [48]. Although the mechanisms underlying fatigue in MS are not yet understood, it seems likely that the widespread presence of abnormal nodes of Ranvier in the NAWM and the resulting inefficient action potential conduction could play a role.

The elongation of paranodal axo-glial junctions, indicated by the paranodal axonal protein Caspr1 in the current study or by its glial counterpart Nf155 [16], suggests that the glial and axonal proteins may have detached from each other, leading to diffusion along the axolemma. Therefore, it could represent a partial or complete detachment of the myelin end loops from the axon and separation of the loops themselves that could result in a complete detachment of the myelin tongues. Although we have not directly demonstrated this using ultrastructural analysis, conditional murine knockouts of the paranodal proteins Caspr1, Nf155, βII spectrin, and 4.1.B [20–24] showed a lack of tight septate junctions and an increased peri-axonal space at the ultrastructural level, which was associated with an elongation of the paranodal protein profiles and dislocation of the juxtaparanodal voltage-gated channels Kv1 towards the PNJ, similar to that seen in MS tissue. This structural change would mean an increment in the width of the peri-axonal space in the paranode and juxtaparanode and, therefore, a progressive change in the membrane capacitance of these compartments. The disruption in the adequate anchorage of K_v_1.2 voltage-gated channels within the juxtaparanode has been shown in previous studies in which the expression of TAG1 and Caspr2, which anchor K_v_1 channels to the axolemma, appeared reduced in NAWM MS tissue [49]. Thus, the partial to complete disruption of the paranode, as well as the possible dissociation of the tripartite juxtaparanodal complex of TAG-1/Caspr2/K_v_1, could explain voltage-gated K_v_1 channel diffusion along the membrane. It is important to note that the disruption to the molecular organisation of the paranodes occurred in the absence of a change in the localisation of Na_v_ channels at the node itself, which is in agreement with our previous studies of demyelinating regions of the MS white matter [47] that showed that changes at the paranodes and movement of K_v_1.2 channels precede alterations at the node, with an altered distribution of nodal proteins only present when paranodes were absent following demyelination. Overall, these histopathological results are especially relevant in progressive MS since paranodal pathology can affect the physiological integrity of axons and can lead to incremental deficits if the inflammatory stimuli persist.

The histopathological analysis of the human tissue showed that a significant proportion of Caspr1-immunopositive elongated paranodes within the MS NAWM tissue were associated with the activated microglia surrounding them. It has been suggested that diffuse axonal injury in the NAWM is closely associated with activated microglia [50–52], and we can now add paranodal pathology as a characteristic of the NAWM changes. The relationship between neuroinflammation and paranodal disruption in myelinated axons was further consolidated by our rat model of meningeal inflammation induced by the chronic expression of LTα and IFNγ. Previous studies have demonstrated the presence of paranodal elongation in spinal cord NAWM regions in a mouse EAE model [16]. However, in this model, the changes in the NAWM were accompanied by the presence of focal demyelinating lesions and occurred over a relatively acute time period. On the contrary, the model used for the current experiments was characterised by widespread microglial activation caused by the chronic presence of pro-inflammatory cytokines over 3 months in the CSF and the absence of demyelinating lesions within the brain. The longitudinal elongation of the Caspr1 expressing paranodal axo-glial junctions and dislocation of the voltage-gated K_v_1.2 channels demonstrated that paranodal disruption can occur in the absence of demyelination. The lack of demyelination also suggests that the paranodal changes occur in the presence of ongoing electrical activity, and it is possible that silent axons might be less vulnerable to the combined effects of TNF and IFNγ, glutamate released by activated glia, and oligodendrocyte dysfunction. Although it was not possible to definitively exclude an influence of anterograde and/or retrograde axonal degeneration that could occur in the absence of demyelination, focal inflammatory mediators released by microglia and astroglia surrounding the axons are likely to play an essential local role. In fact, the extent of both the paranodal elongation and dislocation of K_v_1.2 channels correlated with the number of microglia and astroglia surrounding the altered nodes. It is likely that IFNγ may be playing a major role in this regard as it alone could stimulate glutamate release from microglia, and it has been shown to act on IFNGR receptors on microglia to prime them for phagocytic activity [53], to increase expression of TNFR1 [54] and also to promote various pro-inflammatory responses, including TNF and IL6 release [55].

One of the mechanisms of glial injury at these sites could be the disruption of the axo-glial transport along the myelinic channels of proteins that maintain the adequate structure and function of these junctions [44,56]. Alternatively, the changes to the structure could be caused by the activation of calcium-sensitive proteinases, such as Calpain1, if glutamate levels are not kept at homeostatic levels. Mature oligodendrocytes in the white matter express AMPA/kainate receptors as well as NMDA receptors [27]. However, whereas AMPA/kainate receptors are expressed mainly on the cell soma, NMDA receptors are most highly expressed on myelinating processes [25,26,28], where they presumably play a physiological role in sensing glutamate release by other surrounding cells. Therefore, glutamate released along hemichannels by activated microglia could diffuse into the paranodal and juxtaparanodal peri-axonal spaces and generate paranodal pathology [17] by acting on NMDA receptors in the cytoplasmic channels of the myelin sheath, which include the PNJ and the adaxonal glial membrane [56,57]. Our data suggest that both activated microglia and astroglia release elevated amounts of glutamate in response to stimulation by pro-inflammatory cytokines. The effects of these pro-inflammatory cytokines are highly relevant in the context of MS as recent studies have shown that TNF, IFNγ, and LTα levels are increased in the CSF of progressive MS patients and correlated with meningeal inflammation, cortical demyelination, and activation of MHCII+ microglia [58,59]. In addition, increased CSF levels of TNF and IFNγ can induce endogenous expression of TNF and IFNγ in the underlying cortical parenchyma [38]. TNF and LTα interacting with the TNFR1 receptor can induce glutamate release by microglia via a gap junction/hemichannel system in an autocrine manner [32,33,60] and inhibit glutamate transporters in astroglia by inducing down-regulation of EAAT2/GLT-1 mRNA [34–36]. The increment in length of Caspr1+ paranodes in the organotypic slices treated with TNF and IFNγ confirmed the significant role of these cytokines in the paranodal axo-glial pathology. The findings that both TNF + IFNγ-treated microglial-conditioned medium and glutamate itself could mimic the paranodal changes and that the effects of the cytokines could be inhibited by the noncompetitive NMDA antagonist MK-801 strongly implicate glutamate signaling as a key molecular mediator driving paranodal pathology in the NAWM of the MS brain. Our study did not look at the effects of MK801 alone on the organotypic slice cultures. However, although MK-801 has been reported to induce neurotoxicity when administered chronically at high doses in vivo [61,62], no paranodal myelin damage was observed when isolated spinal cord tissues were treated with MK801 alone at the same dose used in our study [17]. In further support of our experimental results, MRS studies in MS patients have demonstrated increased free glutamate in regions of MS NAWM compared to healthy controls [29–31], and increased glutamate levels have also been found in MS CSF [63]. Our novel result also opens the possibility of therapeutic intervention by reducing microglial/astroglial glutaminase activity, blocking microglial hemichannels (Cx32, Cx36, and Cx43), or by inhibiting TNF and IFNγ signaling.

Our biophysical model simulations of a CNS myelinated axon suggest that the functional consequences of changes at the paranode on AP conduction could be highly detrimental, even if only a small proportion of paranodes along a myelinated axon are disrupted. Recent computational models have simulated paranodal retraction by increasing the nodal width [64] equating the nodal resistance to the paranodal resistance [65] or by reducing the number of myelin loops in the paranode [66]. However, these models only simulated these conditions in a single axon diameter [64,65] or in a few of them [66] and in axons that were usually ≥1 μm. In our simulations, paranodal disruption was progressively generated by an increment in the peri-axonal space width, which led to velocity reduction and conduction failure in small-diameter axons. This is in line with thin axon diameter biophysical theory on saltatory conduction with ion channel delocalization [67] and also stresses the importance of the width of the peri-axonal space [41]. Our data imply that, because axons with small diameter have a lower myelin sheath resistance and a higher axoplasmic resistance, they have a higher dependency on the structural integrity of the PNJ to shunt electrical currents and maintain fast and efficient conduction. Furthermore, these results also highlight the importance of modelling the paranodal and juxtaparanodal compartments, as well as the nodal and internodal compartments, as they are highly sensitive regions that are essential in balancing charge flows with the node of Ranvier. In MS NAWM tissue, the distribution and density of activated microglia and macrophages can vary significantly throughout the WM tracts of the brain and spinal cord [3,16,50,51]. To take this heterogeneity into consideration, we decided to simulate different patterns of disrupted (paranode and juxtaparanode width increment) and healthy nodes to characterise the effect of a highly variable paranodal junction disruption on AP conduction within a myelinated axon, from mildly to severely affected. This showed that conduction failure occurred in axons with a fibre diameter smaller than 0.8 μm with just 5 consecutive disrupted paranodes. Different patterns of disrupted and healthy paranodes made conduction fail at some point along the path in axons with a fibre diameter smaller than 1.1 μm. These simulations not only demonstrate that AP velocity can significantly decrease when the structure of the paranodal axo-glial junction is disrupted but also show that conduction failure of a myelinated axon might depend on the number of consecutive nodes disrupted and the number of healthy nodes in between patches of disrupted nodes. Therefore, they demonstrate that diffuse paranodal axo-glial junction pathology in MS NAWM can significantly alter the efficiency and velocity of AP conduction, and consequently the overall neurological function in MS.

In conclusion, our data strongly suggest that diffuse pathology in the NAWM, which includes paranodal disruption, could be caused by the presence of local cytokine-induced inflammation leading to excess glutamate release from microglia. Such paranodal pathology, which cannot be attributed to focal demyelinating lesions, would be expected to alter the efficiency and velocity of AP conduction, adding to the overall neurological dysfunction in MS. This could also be relevant to white matter changes seen in other neurodegenerative conditions in which chronic microglial activation is a feature.

## Materials and methods

### Human postmortem tissue

Tissue blocks for this study were provided by the United Kingdom Multiple Sclerosis Society Tissue Bank at Imperial College London. Brains were collected following fully informed consent via a prospective donor scheme following ethical approval by the National Multicentre Research Ethics Committee (MREC 02/2/39). Thirty-four tissue blocks (2 × 2 × 1 cm) were selected from 20 cases of neuropathologically confirmed SPMS (Table 1; 11 females) with a mean age of 54.3 yrs (range 38 to 76 y), mean postmortem delay (PMD) of 22 h (range 12 to 48 h), and a mean disease duration of 28.4 y (range 12 to 42 y). Sixteen blocks were also selected from 9 non-neurological control brains (Table 2; 3 females), with a mean age of 73.7 y (range 50 to 88 y) and a mean PMD of 23 h (range 8 to 48 h). All tissue blocks were from the precentral gyrus (containing the primary motor cortex) and the cerebral peduncle located in the midbrain, due to the presence of white matter (WM) tracts that contain highly longitudinally aligned axons that would make quantitative analysis more precise. The blocks were fixed in 4% paraformaldehyde in PBS (4% PFA), cryoprotected in 30% sucrose, frozen in isopentane on dry ice, and stored at −75°C. Cryosections were cut at a thickness of 10 μm and stored at −75°C.

**Table 1:** Demographic data for postmortem MS cases.

Case no	Age (y)	PMD (h)	Gender	Duration (y)	Cause of death
MS404	55	17	F	34	Pneumonia, septicaemia
MS406	62	23	M	42	Pneumonia, MS
MS411	61	24	M	29	Pneumonia, MS
MS422	58	25	M	25	Pneumonia, MS
MS444	49	18	M	20	Renal failure
MS461	43	13	M	21	Bronchopneumonia
MS466	65	25	F	36	Pneumonia, Lung carcinoma
MS478	63	24	F	39	Bowel cancer, MS
MS500	50	32	M	29	Urinary sepsis
MS510	38	19	F	22	Pneumonia
MS517	48	12	F	25	Chest sepsis, MS
MS523	63	20	F	32	Bronchopneumonia, MS
MS530	42	15	M	21	MS
MS535	65	12	F	40	MS
MS542	76	12	F	35	Pneumonia, MS
MS549	50	8	M	29	MS
MS567	45	48	F	23	MS
MS584	42	26	F	12	MS
MS585	53	27	F	27	Bronchopneumonia, MS
MS587	58	36	F	20	Cardiac arrest, MS
***N* = 20**	**mean = 54**	**mean = 22**	**22F:12M**	**mean = 28**	

Age at death, postmortem delay, gender, and duration of disease of the MS cases used in this study. The case numbers represent the UK MS Society Tissue Bank case identifiers. The cause of death is that presented on the death certificates.

MS, multiple sclerosis; PMD, postmortem delay.

**Table 2:** Demographic data for postmortem non-neurological control brains.

Case no	Age (y)	PMD (h)	Gender	Cause of death
C48	68	10	M	Colon cancer
C54	66	16	M	Not available
C72	77	26	M	Pneumonia
C74	84	22	F	Old age
C75	88	8	M	COPD
C76	87	31	M	Pneumonia
PDC29	82	48	M	Liver and lung cancer
PDC39	50	28	F	Renal cancer
PDC40	61	15	F	Ovarian cancer
***N* = 9**	**mean = 74**	**mean = 23**	**5F:11M**	

Age at death, postmortem delay, and gender of the control cases used in this study. The case numbers represent the UK MS Society Tissue Bank (e.g., C48) and UK Parkinson’s Disease Brain Bank case identifiers (e.g., PDC29). The cause of death is that presented on the death certificates.

COPD, chronic obstructive pulmonary disease; PMD, postmortem delay.

**Table 3:** Primary antibody details.

Antibody	Specificity	Dilution	Clone	Source
MOG	Myelin oligodendrocyte glycoprotein	1:50	Mouse (IgG2a) Y10	Reynolds, Imperial
MBP	Myelin basic protein	1:500	Rabbit polyclonal	Merck
NFil	200 kD neurofilament protein	1:500	Mouse (IgG1) NE14	Merck
SMI32	Dephosphorylated neurofilament proteins	1:500	Mouse (IgG1) SMI32	BioLegend
Caspr1	Contactin-associated protein 1	1:300	Rabbit EPR7828	Abcam
Caspr1	Contactin-associated protein 1	1:500	Mouse (IgG1) K65/35	Neuromab
Pan-Na_v_	Voltage-gated sodium channels	1:100	Mouse (IgG2a) K58/35	Neuromab
Kv 1.2	Voltage-gated potassium channel 1.2	1:100	Mouse (IgG2a) K14/16	Neuromab
HLA-DR	MHC class II human leucocyte antigen DR	1:200	Mouse TAL.1B5	Dako Agilent
IBA1	Ionized calcium binding adaptor molecule 1	1:500	Rabbit polyclonal	Wako
GFAP	Glial fibrillary acidic protein	1:500	Rabbit polyclonal	Dako Agilent
Calbindin1	Calbindin1 (Purkinje neurons)	1:500	Mouse (IgG1) CB-955	Merck
NeuN	Neuron specific nuclear protein	1:500	Mouse (IgG1) A60	Merck

Antigen, specificity, concentration, clone, and source of the primary antibodies used in this project.

### Primary and secondary antibodies

The primary antibodies used in this project were: mouse anti-MOG (clone Y10, Prof R Reynolds, Imperial College London, UK); rabbit anti-myelin basic protein (MBP) (Polyclonal, Merck, Darmstadt, Germany); mouse anti-neurofilament-H protein (clone NE14; Merck, Darmstadt, Germany); mouse anti-dephosphorylated neurofilament protein (clone smi32; Biolegend, San Diego, California, United States of America); rabbit anti-caspr1 (clone EPR7828, Abcam, Cambridge, UK); mouse anti-caspr1 (clone K65/35; Neuromab, Davis, California, USA); mouse anti-pan-Na_v_ channels (clone K58/35, Neuromab, Davis, California, USA); mouse anti-K_v_1.2 channels (clone K14/16, Neuromab, Davis, California, USA); mouse anti-HLA-DR (clone TAL.1B5, Dako Agilent, Santa Clara, California, USA); rabbit anti-IBA1 (Polyclonal IgG, Wako Pure Chemical Corporation, USA); rabbit anti-Glial Fibrillary Acidic Protein (GFAP) (Polyclonal, Dako Agilent, Santa Clara, California, USA); mouse anti-Calbindin1 (clone CB-955, Merck, Darmstadt, Germany); and mouse anti-NeuN (clone A60, Merck, Darmstadt, Germany) (Table 3). All the secondary antibodies used for immunofluorescence were purchased from ThermoFisher Scientific (USA): Alexa Fluor 546 Goat Anti-Mouse IgG (H+L), Alexa Fluor 546 Goat Anti-Rabbit IgG(H+L), Alexa Fluor 488 Goat Anti-Mouse IgG (H+L) Fluor 488 Goat Anti-Rabbit IgG (H+L), Alexa Fluor 488 Goat Anti-Mouse IgG1, Alexa Fluor 546 Goat Anti-Mouse IgG1, Alexa Fluor 647 Goat Anti-Mouse IgG1, Alexa Fluor 488 Goat Anti-Mouse IgG2b, Alexa Fluor 647 Goat Anti-Mouse IgG2b, Alexa Fluor 488 Streptavidin, Alexa Fluor 546 Streptavidin, and Alexa Fluor 647 Streptavidin. For immunohistochemistry, the following biotinylated antibodies were used from Vector Laboratories (UK): biotinylated anti-rabbit IgG (H+L) made in goat, biotinylated anti-mouse IgG (H+L) made in horse, and biotinylated anti-mouse IgG2a made in goat (Life Technologies, ThermoFisher Scientific).

### Immunostaining on postmortem human tissue

For immunofluorescence for all antigens, sections were fixed with 4% PFA for 30 min (Table 3), except for the Pan-Na channel antibody, for which fixation was not longer than 10 min. After fixation, sections were post-fixed with 100% methanol at −20°C (Sigma) for 10 min and washed in 0.1 M PBS-0.3% Triton X-100 (Sigma-Aldrich) 3 times, 5 min each. After post-fixing, sections were blocked and permeabilised with 0.1 M PBS containing 10% normal horse/goat serum (Sigma-Aldrich) and PBS-0.3% Triton X-100 for 1 h at room temperature. Finally, sections were incubated overnight at 4°C in a humid chamber with primary antibodies (Table 3) in 0.1 M PBS containing 10% normal horse/goat serum and PBS-0.3% Triton X-100 (Sigma-Aldrich). After primary antibody incubation, sections were thoroughly rinsed in 0.1 M PBS at least 3 times, 5 min each. After rinsing, sections were incubated with the appropriate secondary antibody conjugated to biotin for 1 h for MOG, SMI32, and Pan-Nav antigens and rinsed in 0.1 M PBS. After rinsing, sections were incubated with Alexa Fluor Streptavidin or the appropriate species-specific secondary fluorochrome conjugated antibody for 2 h at room temperature. Finally, the tissue was rinsed in 0.1 M PBS and dH_2_O, nuclei counterstained with DAPI (diluted in 1:2000, Sigma-Aldrich), and mounted with Vectashield Antifade Mounting Media (Vector Laboratories). The coverslips were fixed to the slides with clear nail polish.

Immunohistochemistry was performed for HLA-DR antigen with the ImmPRESS HRP Anti-Mouse IgG (Peroxidase) Polymer Detection Kit, made in horse (Vector Laboratories). Tissue endogenous peroxidase activity was blocked with the Bloxall Blocking solution (SP-6000) for 10 min and then incubated with 2.5% horse serum for 20 min. After blocking and washing with 0.1 M PBS, the primary antibody was incubated overnight at 4°C in a humid chamber. The ImmPRESS Polymer Reagent was incubated at room temperature for 30 min, and the signal was developed with ImmPact DAB (SK-4105, Vector Laboratories). The tissue was then rinsed with tap water for 5 min to stop the reaction. Slides were counterstained with haematoxylin (Sigma-Aldrich) for 5 min, washed with tap and distilled water, dehydrated with washes of 70%, 90%, and 100% of ethanol (2 min each), cleared with xylene for 10 min and mounted with DPX mounting medium (Sigma-Aldrich).

### Lentiviral vector production

Lentiviral (LV) vectors expressing the human LTα (LVLTα), human IFNγ (LVIFNγ), or enhanced green fluorescent protein (LVGFP) genes were produced exactly as described previously [38], using the human immunodeficiency virus type 1 (HIV-1) transfer vector (pRRL-sincppt-CMV-eGFP-WPRE genome plasmid) with a human cytomegalovirus promoter (CMV) promotor. Complementary DNA sequences (cDNA) for human LTα or IFNγ were codon optimised for rat, including a 5′ Kozak sequence, and synthesised by Gene Art (Life Sciences, Paisley, UK). The biological and physical titres of the purified and concentrated vectors were calculated as described previously [38]. The LV genome copy number was calculated using the Clontech Lenti-X qRT-PCR Titration kit (Takara, Saint-Germain-en-Laye, France).

### Stereotaxic surgery and tissue processing

Eleven 8- to 10-week-old female Dark Agouti (DA) rats (140 to 160 g) were obtained from Janvier (France) and kept in groups of 3 to 4 in a 12-h light/dark cycle with food and water provided ad libitum. Stereotaxic injections of LV vectors into the subarachnoid space were carried out at 0.9 mm caudal to bregma, in the midline, following previously published methods [38,68]. Rats were either naive with no treatment or were injected once with incomplete Freund‘s Adjuvant (IFA). Rats were induced and maintained under general anaesthesia for the entire procedure using 2% isoflurane (Abbott Laboratories, Berkshire, UK, and oxygen 2 l/min), their scalps were shaved and disinfected with Videne antiseptic solution (Ecolab, Northwich, United Kingdom). Subcutaneous injections of 0.9% saline (Sigma) and 0.01 mg/kg buprenorphine (Vetergesic; Alstoe Animal Health, North Yorkshire, UK) were performed to provide post-surgery rehydration and analgesia. Rats were positioned on the stereotaxic frame (Stoelting, Dublin, Ireland), and an incision was made through the scalp to visualise the skull bregma. A 2 mm diameter hole in the skull was drilled at the midline at 0.9 mm caudal to bregma, at the level of the motor cortex. Injections were performed with a finely calibrated glass capillary attached to a 26-gauge needle of a 10 μl Hamilton syringe (Hamilton, Graubunden, Switzerland). The needle was inserted to a depth of 2.4 mm below the dural membrane. Four μl of LV vector preparation (2 μl of each vector), diluted in TSSM with 0.5 mM monastral blue tracer, was introduced at a rate of 0.2 μl/min using an automated infuser (KD Scientific, USA). Viruses were injected at a total of 1 × 10^12^ genomic copies/μl (GC/μl) for LTα and GFP and 1 × 10^10^ GC/μl for IFNγ. The needle was left in place for 5 min to allow diffusion of the sample from the area of injection, then withdrawn and the incision was sutured (Mersilk; Covidien, Ireland). The sutures were removed after 7 to 10 days, and the animals were monitored daily.

At the termination of the study, rats received an overdose of sodium pentobarbital (200 mg/ml Euthatal; Merial Animal Health, Essex, UK) by intraperitoneal injection. Rats were perfused with 50 ml PBS followed by 100 ml 4% PFA in PBS through the left ventricle at 90 days post vector injection. Brains were removed and post-fixed in 4% PFA (4 h, room temp), prior to cryoprotection in 30% sucrose solution in PBS (48 h or until equilibrated). Brains were embedded in optimal cutting temperature compound (OCT; Tissue-Tek; Sakura, the Netherlands), frozen in isopentane on dry ice and sectioned at 10 μm in the coronal plane throughout the brain. The rat tissue sections were stained following the same protocol as the human tissue except the initial 4% PFA fixation step.

### Primary rat microglial and astroglial cultures

P0-P2 Sprague Dawley rats (Charles River Laboratories, USA) were decapitated following the UK Home Office regulations. The brains were isolated, and the cerebral cortices were dissected with sterile autoclaved tweezers and dissecting scissors and freed of meninges to avoid any fibroblast contamination. The cortices of 3 pups were transferred to a 50 ml sterile polypropylene conical tube with dissection medium, which contained Hank’s Balanced Salt Solution (HBSS 1X, Gibco, ThermoFisher Scientific). The supernatant was removed and replaced by digestion mix containing Minimum Essential Media (MEM with 4 mM glutamine, Gibco, ThermoFisher Scientific), 2.5 mg/ml Papain (14 units/mg, Sigma-Aldrich, Merck), 40 g/ml DNAse (980 Units/mg, Sigma-Aldrich, Merck), and 240 g/ml l-Cysteine (Sigma-Aldrich, Merck). The 50 ml Falcon tubes were placed in a water bath (Fisher Scientific) at 37°C for 1 h; every 15 min, the tissue was dissociated by gentle pipetting. After 1 h, the cortices were resuspended in 30 ml warm dissection medium, filtered through a 70 μm nylon cell strainer (Corning Incorporated, USA) to remove any non-dissociated fragments and centrifuged at 500 rpm for 5 min. The supernatant was discarded and the pellet resuspended in 15 ml of culture medium. The culture medium was Dulbecco’s Modified Eagle Medium Nutrient Mixture F-12 (DMEM F12, Gibco, ThermoFisher Scientific) supplemented with 5 ml streptomycin-penicillin (10,000 U/mL, Gibco, ThermoFisher Scientific), 5 ml L-glutamine (200 mM, Gibco, ThermoFisher Scientific), and 50 ml heat-inactivated fetal bovine serum (Sigma, Merck). The mixture of dissociated cells was plated in a T75 culture flask (Nunc EasyFlask 75 cm^2^ cell culture flasks, Thermo Scientific), previously coated with poly-D-lysine hydrobromide (PDL, Sigma, Merck) for 2 h and washed with sterile water 3 times. The mixed glial cultures were maintained for 1 week in a humidified incubator at 37°C and 5% CO_2_ for 1 week, and half the medium was changed every 2 days to replace growth factors. After a week, astroglia formed a connected confluent dense monolayer in the bottom of the flask, whereas the majority of the microglia were floating. Primary microglial cells were isolated from the astroglial cell bed by mechanical agitation by vigorously tapping the flasks. Subsequently, the cells were plated in a 24-well plate (Nucleon Delta Surface, Thermo Scientific) at a cell density of 5 × 10^4^ cells/well. After 24 h, microglia were attached to the bottom of the plate, and they were treated with the pro-inflammatory cytokines. Primary astroglial cultures were prepared from the confluent layer of astroglia remaining on the bottom of the flask. Cells were detached with Accutase solution (Sigma-Aldrich) and replated at 5 × 10^5^ cells per well into PDL-coated 24-well plates.

### Cerebellar organotypic tissue cultures

P8/P9 Sprague Dawley rats (Charles River Laboratories) were decapitated following the UK Home Office regulations. The hemispheres were separated and plated in a petri dish containing a sterile filter paper and cold dissection medium (DMEM), supplemented with 5 ml of streptomycin-penicillin (10,000 U/mL, Gibco, ThermoFisher Scientific). Hemispheres were mounted with Superglue together with a rectangular piece of sterile solid agar (4 g of agar (Sigma) in 200 ml of distilled water) and sliced using a Leica VT1200s vibratome in the parasagittal plane at 400 μm thickness. The cerebellar slices were transferred into a 60 mm petri dish containing cold medium until the whole hemisphere was sliced. Later, they were plated on sterile Biopore PTFE membranes of 0.4 μm pore size (Millicell-CM culture inserts, Merck) in a sterile 6-well plate (Costar, Corning Incorporated) with 1 ml of nutrient medium underneath every culture insert. The nutrient medium contained 200 ml Neurobasal-A-medium (Gibco, ThermoFisher Scientific) and 100 ml Hank’s Balanced Salt Solution (HBSS 1X, Gibco, ThermoFisher Scientific) supplemented with 5 ml streptomycin-penicillin (10,000 U/mL, Gibco, ThermoFisher Scientific), 5 ml L-glutamine (200 mM, Gibco, ThermoFisher Scientific), 4.4 ml D-glucose (200 g/L, Gibco, ThermoFisher Scientific), and 2 ml vitamin B27 Plus Supplement (50X, Gibco, ThermoFisher Scientific). One to two cerebellar slices were plated per insert and maintained in an incubator (HeraCell Vios 160i, ThermoFisher) at 37°C, 5% CO_2_ for 9 to 10 days, replacing half the medium every other day to replace the growth factors. In order to check the integrity of the slices, the macroscopic structure was checked with an inverted microscope (Olympus CKX53). They were also stained with the cell integrity marker propidium iodide (PI, Molecular Probes). PI was added at a concentration of 5 μg/ml, incubated for 60 min in the culture medium and imaged with the inverted fluorescence microscope. After 9 to 10 days, in vitro (DIV) healthy slices flattened to approximately 100 μm and were selected for further experiments.

### Cerebellar and glial culture treatment

The cerebellar slices were treated either 3 times 24 h apart with combinations of recombinant TNF (recombinant rat TNF, Biolegend), LTα (recombinant human LT-α, Abcam), and IFNγ (recombinant rat IFNγ, Biolegend) at 50 ng/ml or twice 24 h apart at a concentration of 100 ng/ml (glutamate levels were always measured 24 h after the last dose). The microglial cultures were treated either once or twice with doses of 100 ng/ml or 200 ng/ml of TNF, IFNγ, LT-α, TNF + IFNγ, and LT-α + IFNγ (each dose was administered every 24 h, and glutamate levels were always measured 24 h after the last dose). The cerebellar cultures were also treated with conditioned microglial medium after two 100 ng/ml doses of TNF + IFNγ (in this case, each well contained 800 μl of conditioned medium and 200 μl of nutrient medium). In addition, the cerebellar slices were treated directly with glutamate (L-Glutamic acid, Sigma-Aldrich, Merck) twice every 24 h at a concentration of 75 μM and 100 μM. A group of slices treated with 100 ng/ml of TNF + IFNγ were also incubated at the second dose with 0.6 mM of MK-801 maleate [17], which is a noncompetitive NMDA antagonist (ab120028, Abcam). The culture medium was replaced entirely when the cytokines or glutamate were added to the primary microglia or to the tissues.

Primary astroglia were treated 24 h after plating with 100 or 200ng/ml of recombinant rat TNF (Biolegend) and recombinant rat IFNγ (BioLegend). For glutamate release assays, cells were incubated in ABM astrocyte Basal medium supplemented with AGM astrocyte growth medium Bulletkit for 24 h, while for glutamate uptake assays, 100 μM glutamate was added. Culture supernatant was collected and glutamate concentration in the medium was measured as described below. Glutamate uptake by astrocytes was measured by subtracting the amount of glutamate measured in the medium from the amount initially added to the cells.

### Immunofluorescence of organotypic cerebellar slice cultures

Immunofluorescence analysis of the cerebellar slices was carried out as previously published [69]. The cultures were fixed with cold 4% PFA (1 ml added on top of the filter and 1 ml underneath) for 1 h at room temperature. After fixation, sections were post-fixed with 20% methanol and washed in 0.1 M PBS-0.3% Triton X-100. For further permeabilisation, slices were incubated with 0.1 M PBS-0.3% Triton X-100 overnight. After permeabilisation, slices were blocked with 20% bovine serum albumin (BSA, Sigma, Merck) and PBS-0.3% Triton X-100 for a minimum of 4 h at room temperature. Slices were removed from the insert and incubated overnight with primary antibodies (Table 3) at 4°C in a humid chamber. After primary antibody incubation, sections were thoroughly rinsed in 0.1 M PBS, 3 times 10 min each and incubated with the appropriate secondary antibody conjugated to the appropriate fluorochrome for 4 h at room temperature. The tissue was rinsed in 0.1 M PBS (3 times, 10 min each) and dH_2_O, nuclear counterstained with DAPI (diluted in 1:2000), and mounted on slides with Vectashield Antifade Mountant. Coverslips (24 × 50 mm, VWR, International) were fixed to the slides with clear nail polish.

### Glutamate colorimetric assay

The glutamate concentration in the conditioned medium of primary microglial cultures was assayed with the glutamate colorimetric assay kit from Abcam (no. 83389), according to the manufacturer’s instructions. The samples were incubated at 37°C for 30 min, and the absorbances read at 450 nm using a SPARK multimode reader (Tecan, Männedorf, Switzerland). The glutamate concentration was extrapolated from the standard curve regression equation and the absorbances corrected for background values. Results were expressed in μM and represented by the mean ± standard error of the mean (SEM) of 3 to 4 duplicates per condition.

### Data analysis

All the data were processed in R (R Project for Statistical Computing) and plotted with the package ggplot2. For the human tissue data, the rat tissue data, and the organotypic tissue cultures, the nonparametric Mann–Whitney test was used to compare groups and nonparametric Spearman rank correlation test for correlation between variables. For the rat tissue data, the Kruskal–Wallis test was also used to compare across groups. For the microglial culture studies, the nonparametric Friedman rank sum test was used to compared across treatment groups and timings, and Wilcoxon pairwise comparison with a Bonferroni correction was used to compare between groups (*p < 0.05, **p < 0.01, ***p < 0.001, ****p < 0.0001). Mean ± SEM per group was calculated in all the cases, and box plots (showing the median, the 25% to 75% quartiles, the minimum and the maximum), bar graphs, or point graphs were plotted.

### Image acquisition and analysis

Images of immunostained sections from the postmortem human tissue and the rat tissue were obtained with an epifluorescence Olympus BX63 scanning microscope or a SP8 Leica confocal microscope. The cerebellar organotypic cultures were imaged exclusively with the confocal microscope due to their thickness. For the latter, a range of 4 to 6 z-stacks were taken with an average thickness of 15 μm with a step size of 0.3 μm. All images were analysed using Fiji (Image J, NIH, USA) and prepared in Illustrator (Adobe Systems). The quantification of all the cases/samples was performed with the observer blinded to case identification. Regions of interest (ROIs) in human MS tissue were defined as NAWM regions at least 4 mm away from a focal demyelinating lesion. MOG immunofluorescence images were taken at 4× to get an overall scan area of the whole block to select NAWM regions. In the postmortem human tissue, 10 images of HLA-DR+ staining from NAWM regions were taken at 20×. Microglial activation was analysed by quantifying the area occupied by HLA-DR+ microglial labelling microglia by thresholding the images to a specific intensity per image acquired and dividing them by the total area of each image. In the rat tissue, the number of IBA1+ microglia and GFAP+ astroglia were counted in images taken at 60× magnification.

PNJ disruption was analysed by measuring the length of the axonal paranodal protein Caspr1 immunofluorescence staining in the postmortem human tissue, rat tissue, and cerebellar organotypic tissue slices. Caspr1, Pan-Na, K_**v**_1.2, and Caspr1-SMI32 triple and double immunofluorescent images were captured with a 63× oil immersion objective. Only Caspr1 positive axons that were in focus were measured in 10 ROIs in the human and rat tissues. In the cerebellar organotypic slices, the ROIs corresponded to a range of 4 to 6 z-stacks located in the regions with a high density of Purkinje cell axons. In rat sections and cerebellar organotypic tissue slices, 200 focused Caspr1-stained paranodes were measured in each preparation. In the postmortem human tissue, a total of 6,800 Caspr1-stained paranodes from 34 blocks of 10 pathologically confirmed cases and 3,200 Caspr1-stained paranodes from 16 blocks of 10 control cases were analysed. In the rat tissue, 1,000 Caspr1-stained paranodes from the LT-α + IFNγ group, 600 from the GFP group, and 600 from the naïve group were analysed. In the cerebellar organotypic tissue slices, we analysed 1,200 Caspr1-stained paranodes from 6 cerebellar slices treated with 50 ng/ml of TNF + IFNγ, 800 Caspr1-stained paranodes from 2 cerebellar slices treated with 100 ng/ml of TNF + IFNγ, 800 Caspr1-stained paranodes from 4 control cerebellar slices, 1,000 Caspr1-stained paranodes from 5 cerebellar slices treated with microglial conditioned medium, 800 Caspr1-stained paranodes from 4 cerebellar slices treated with 75 μM glutamate, 400 Caspr1-stained paranodes from 4 cerebellar slices treated with 100 μM glutamate, and 1,000 Caspr1-stained paranodes from 5 cerebellar slices treated with TNF + IFNγ and MK-801.

For quantification of voltage-gated K_**v**_1.2 and Na_**v**_ channel dislocation, Caspr1-K_**v**_1.2 and Caspr1-Na_**v**_ double fluorescent images were taken with the 60× oil immersion objective for each human and rat sample. Only positive Caspr1-K_**v**_ 1.2 and Caspr1-Pan Na_**v**_ axons were studied, in 10 ROIs, with a minimum of 50 axons per tissue block. RGB line intensity profiles of Caspr1, K_**v**_ 1.2, and Pan Na_**v**_ were obtained along the length of the stained axon to assess K and Na channel dislocation. An overlapping region between 2 signals was defined as the axonal area where K_**v**_ or Na_**v**_ channels were located in the paranodal axolemma. Using RGB intensity line profiles acquired with Fiji, intensity signals of K_**v**_1.2 or Na_**v**_ and Caspr1 were subtracted from one another per axon. If the difference between both signals was smaller than a variable threshold at the same point in distance, an overlapping region was confirmed. Moreover, the total proportion of axons with overlapping regions was measured in MS and non-neurological human tissue and in the GFP, LTα-IFNγ, and naive rat tissue. The means ± SEM of all line profiles were calculated per case and across groups (the code used to calculate channel dislocation can be found in S1A Text).

### Computational modelling

A double cable core model composed of 21 nodes was built and solved numerically in NEURON [70] using the backward Euler implicit integration method [40]. The equilibrium potential was set to −80 mV, and the simulations were run at a temperature of 37°C. The code used to generate this model can be found in the S1B Text. The geometrical and biophysical parameters used in this model are summarised in Fig 7B and 7C. Simulations across the 7 different diameters (d _core_ = 0.4, 0.6, 0.8, 1, 1.2, 1.4, 2.7 [μm]) were initiated and allowed to reach the resting potential for 1 ms before a current of 2 nA was injected in the midpoint of node 1 as a square pulse of 0.1 ms duration. The relationship between the core diameter, fibre diameter, and myelin sheath thickness was taken from EM studies of the macaque brain [42]. The number of myelin sheath was calculated from the myelin periodicity value of 0.0156 μm [71]. The node to node length was taken from the linear relationship measured from rat nerve fibres (117.52 + 30.47 * d_fibre_) [72]. The juxtaparanodal length was extrapolated from the diameter-dependent scaling relationship from the ventral root of cats (19.6 + 2.58 *d_fibre_) [73]. The paranodal length was determined from the average value of Caspr1 staining from the non-neurological control tissue, and the internodal length was derived from the subtraction of 2 paranodes and juxtaparanodes lengths from the node-to-node length across the 7 diameters. The nodal length (1 μm) was kept constant for simplicity reasons. Lastly, the peri-axonal space dimensions of the paranode, juxtaparanode, and internode were based on the data measured by following dextran tracers in myelinated fibres of mouse sciatic nerves [74]. The propagation of the AP along the axon was measured up to 10 ms (2,000 points plotted/ms), and measurements were recorded at nodes 4 and 16 AP, and the AP amplitude, width, and conduction velocity were measured. AP amplitude [mV] was described as the voltage difference between the most negative voltage during the hyperpolarisation afterpotential and action potential peak (V_max)_. AP width [ms] was defined as the time difference at half the amplitude, and conduction velocity [m/s] as the distance between V_max_ of the spikes at node 4 and 16. We used 4 types of voltage-gated channels, which were as follows: Fast Na_v_ channels, Persistent Na_v_ channels, Slow Persistent K_v_ channels, and Fast K_v_ channels and Leakage channels. The following voltage-gated channel characteristics were used in our model:

The maximum nodal Na_v_ channel density was set to 1,000 channels/μm^2^ [75,76] and the single conductance of a fast Na_v_ channel set to 15 pS [77]. Thus, the maximal conductance was 1.5 S/cm^2^. Fast Na_v_ channel gating was based on the gating dynamics from measurements of a human nerve used by Schwarz and colleagues [78] at 20°C. The following gating dynamics were based on these experimental data, and the temperature change was adjusted with a q10 = 2.2 [39,40,79].

I_Na_ = g_Na_ * m^3^ * h * (V_m_ − E_Na_)

α_m_ = 6.57(V_m_ + 20.4)/(1 − exp(−(V_m_ + 20.4)/10.3) β_m_ = 0.304(−(V_m_ + 25.7)/(1 − exp((V_m_ + 25)/9.16) αh = 0.34(−(V_m_ + 114))/(1 − exp((V_m_ + 114)/11) βh = 12.6/(1 + exp(−(V_m_ + 31.8)/13.4)

Persistent Na_v_ channel conductance was based on the data from rat ulnar nerves [80]. The estimated density was 6.5 channels/μm^2^ with a conductance of a single channel of 20 pS. Thus, the maximum conductance in this model was set to 0.01 S/cm^2^. This value was taken from previous myelinated axon computational studies [39,40]. The membrane gating dynamics used were the same as previous computational models [39,40,81].

I_Nap_ = g_Nap_ * p^3^ * (V_m_ − E_Na_)

α_p_ = 0.0353(V_m_ + 27)/[1 − exp(−(V_m_ + 27)/10.2]

β_p_ = 0.000883(−(V_m_ + 34))/[1 − exp((V_m_ + 34)/10]

Slow K_v_ channels have an estimated density of 110/μm^2^ [82]. From human nerve electrophysiological studies, the single conductance of a channel was quantified to be between 7 and 10 pS [76,82,83]. In this model, the single conductance of a slow K_v_ channel was set to 8 pS. Thus, with a density of 110 channels/μm^2^ and the maximum conductance was 0.088 S/cm^2^. The gating dynamics were based on the model of McIntyre and colleagues [40].

I_Ks_ = g_Ks_ * s * (V_m_ − E_k_)

α_s_ = 0.3/(1 + exp((V_m_ + 53)/5))

β_s_ = 0.03/(1 + exp((V_m_ + 90)/ − 1))

The density of fast K_v_ channels was estimated to be 12 channels/μm^2^ [82], while the single conductance of a channel measured in the rat peripheral nerve was 17 pS [82,84]. Thus, the maximum conductance in our model for the fast K_v_ channels was set to 0.02 S/cm^2^. These values have been used in previous computational studies (q10 = 3) [40,82].

I_k f_ = g_k f_ * n^4^ * (V_m_ − E_k_)

α_n_ = 0.00798(V_m_ + 93.2)/(1 − exp(−(V_m_ + 93.2)/1.1))

β_n_ = 0.092(−(V_m_ + 76))/(1 − exp(−(V_m_ + 76)/10.5))

The leak conductance of the node was set to gl = 0.007 S/cm^2^ [80], while the leak conductance of the paranode was gl = 0.0005 S/cm^2^, the juxtaparanode and internode conductances were gl = 0.005 S/cm^2^ [85].

### Ethical permissions

All human postmortem tissues used in this study were from brains collected by the UK MS Society Tissue Bank at Imperial College following fully informed consent via a prospective donor scheme under ethical approval by the UK National Multicentre Research Ethics Committee (MREC 02/2/39). All animal experiments were carried out under the regulations of the Animals (Scientific Procedures) Act 1986 of the UK Home Office (Licence 7213) and were given approval by the Central Animal Welfare and Ethical Research Board (cAWERB) of Imperial College London.

## Supporting information

S1 Fig Paranodal Length of MS and non-neurological control blocks. Box plots representing the distributions of paranodal length from NAWM MS and non-neurological control tissue per case. In the *y* axis, the indexes “cbp” correspond to the cerebral peduncle blocks, while the indexes “pcg” correspond to the precentral gyrus blocks. MS, multiple sclerosis; NAWM, normal-appearing white matter. Data and code to reproduce this figure can be found at: https://github.com/PatGal2020/PLOS_submission.
(TIF)

S2 Fig The location of nodal Nav channels was not disrupted in MS NAWM tissue. (A) Confocal images of a double immunofluorescence of Caspr1-stained paranode and nodal voltage-gated Nav channels with the RGB intensity profile of both immunofluorescence signals across the nodal and paranodal compartments. (B) Caspr1 signal was subtracted from Nav, and when the difference between them was smaller than a variable Intensity Threshold, that point was considered an overlapping region. For every threshold calculated, the proportion of overlapping regions was very similar in both groups. MS, multiple sclerosis; NAWM, normal-appearing white matter. Data and code to reproduce this figure can be found at: https://github.com/PatGal2020/PLOS_submission.
(TIF)

S3 Fig Rat model of meningeal inflammation induced by the chronic exposure to LTα/IFNγ. (A) Immunofluorescent image of a coronal rat section stained with MOG. Lentiviral vectors encoding LT-α and IFN-γ genes were injected into the subarachnoid space in the midline of the brain. The white rectangles are a representative of the 10 selected ROIs at the corpus callosum, cingulum, and external capsule. (B) Table of the number of animals used: 5 rats were injected with LTα/IFNγ, 3 rats with GFP, and 3 naives. (C) Immunofluorescent image of a coronal rat section stained with IBA1 and treated with LT-α and IFN-γ. (D) Caspr1-SMI32 immunofluorescence in LT*α*/IFN*γ*, GFP, and naive rat tissue. Confocal images of Caspr1-stained paranodes (red) and SMI32+ axons (green). GFP, green fluorescent protein; IFNγ, interferon-γ; LTα, lymphotoxin-α; MOG, myelin oligodendrocyte glycoprotein; ROI, region of interest. Data and code to reproduce this figure can be found at: https://github.com/PatGal2020/PLOS_submission.
(TIF)

S4 Fig Primary rat microglial cultures images treated with the pro-inflammatory cytokines IFNγ, TNF, and LTα. (A) Timeline diagram showing the timings of the experiments. Microglia were either treated with 1 dose of cytokines or 2 doses 24 h apart. The glutamate in the supernatant was analysed in both cases after 24 h and 48 h. (B) Mean ± SEM glutamate levels from replicates showing the statistical difference between controls and the cytokine treatments at different concentrations: 200 ng/ml (*n* = 3 Control, *n* = 3 TNF, *n* = 3 IFNγ, *n* = 3 TNF + IFNγ), (C) 100 ng/ml (*n* = 3 Control, *n* = 3 LTα, *n* = 3 IFNγ, *n* = 3 LTa + IFNγ), and (D) 2 acute treatments with 100 ng/ml (*n* = 3 Control, *n* = 3 LTα, *n* = 3 IFNγ, *n* = 3 LTα + IFNγ). Nonparametric Friedman test was performed across cytokine groups and timings and post hoc paired-wised Wilcoxon tests to compare groups (* *p* < 0.05, ** *p* < 0.01). IFNγ, interferon-γ; LTα, lymphotoxin-α; TNF, tumour necrosis factor. Data and code to reproduce this figure can be found at: https://github.com/PatGal2020/PLOS_submission.
(TIF)

S5 Fig Glutamate release and uptake by TNF/IFNγ-activated astrocytes. (A) Glutamate release by primary astrocyte cultures treated with TNF/IFNγ (100 ng/ml and 200 ng/ml) after 24 h. (B) Glutamate uptake by primary astrocyte cultures treated with TNF/IFNγ (100 ng/ml and 200 ng/ml) and 100 μM of glutamate after 24 h. Mean ± SEM for glutamate levels from replicates showing the statistical difference between controls and the cytokine treatments. Nonparametric Mann–Whitney test was performed across cytokine groups. IFNγ, interferon-γ; TNF, tumour necrosis factor. Data and code to reproduce this figure can be found at: https://github.com/PatGal2020/PLOS_submission.
(TIF)

S6 Fig Cerebellar organotypic tissue cultures. (A) Image of a live flattened cerebellar slice. The slices were cut at 400 μm thickness and after 8–10 DIV healthy slices flatten to approximately 100 μm. (B, C) Bright field images of cerebellar slices on culture inserts. (D) Confocal image of a cerebellar slice stained with antibodies against Calbindin+ for Purkinje cells and GFAP+ for astroglia. (E) Confocal image of a cerebellar slice stained with antibodies to MBP for myelin and Calbindin for Purkinje cells. (F) Confocal image of a cerebellar slice stained with Caspr1 antibodies. (G) Confocal image of a cerebellar slice stained with SMI32 antibodies. (H) Cerebellar slices were treated with the pro-inflammatory cytokines TNF/IFNγ (3 doses of 50 ng/ml (*n* = 3), 2 doses of 100 ng/ml (*n* = 4)), microglial-conditioned medium (2 doses of the medium from microglia treated with 2 acute doses of 100 ng/ml of TNF/IFNγ), and glutamate (2 doses of 75 mM or 100 mM). IFNγ, interferon-γ; MBP, myelin basic protein; TNF, tumour necrosis factor. Data and code to reproduce this figure can be found at: https://github.com/PatGal2020/PLOS_submission.
(TIF)

S7 Fig The proportion of disrupted paranodes required for conduction failure. The difference in the proportion of disrupted paranodes within an axon of dcore of 0.6 μm can provoke conduction failure and a variable degree of velocity reduction. (A) In axon model of 0.6 μm core diameter, conduction failure occurred when 5 consecutive nodes were disrupted (orange), and the paranodal and juxtaparanodal peri-axonal space widths were increased up to 0.022 and 0.22 μm, respectively. (B) Velocity decay and conduction failure of this axon model under different patterns of disruption (orange means disrupted node, purple, healthy node, and red denotes conduction failure).
(TIF)

S8 Fig The proportion of disrupted paranodes required for conduction failure. The difference in the proportion of disrupted paranodes within an axon of dcore of 0.8 μm can provoke conduction failure and a variable degree of velocity reduction. (A) In an axon model of 0.8 μm diameter, conduction failure occurred when 11 consecutive nodes were disrupted (orange), and the paranodal and juxtaparanodal peri-axonal space widths were increased up to 0.022 and 0.22 μm, respectively. (B) Velocity decay and conduction failure of this axon model under different patterns of disruption (orange means disrupted node, purple means healthy node, and red denotes conduction failure).
(TIF)

S1 Text (A) Algorithm for the quantification of Caspr1-Kv or Caspr1-Na overlapping signals. (B) Code for the computational model generated in NEURON.
(DOCX)

## Abbreviations

AP: action potential
BBB: blood–brain barrier
BSA: bovine serum albumin
CARS: coherent anti-Stokes Raman scattering
cDNA: complementary DNA
CMV: cytomegalovirus
CNS: central nervous system
CSF: cerebrospinal fluid
DA: Dark Agouti
DMEM: Dulbecco’s Modified Eagle Medium
EM: electron microscopy
GFAP: Glial Fibrillary Acidic Protein
GFP: green fluorescent protein
HBSS: Hank’s Balanced Salt Solution
HIV-1: human immunodeficiency virus type 1
IFA: incomplete Freund‘s Adjuvant
IFNγ: interferon-γ
LTα: lymphotoxin-α
LV: lentiviral
MBP: myelin basic protein
MEM: Minimum Essential Media
MOG: myelin oligodendrocyte glycoprotein
MRS: magnetic resonance spectroscopy
MS: multiple sclerosis
NAWM: normal-appearing white matter
Nf155: neurofascin 155
NMDA: N-methyl-D-aspartate
OCT: optimal cutting temperature
PDL: poly-D-lysine
PI: propidium iodide
PMD: postmortem delay
PNJ: paranodal junctions
ROI: region of interest
SEM: standard error of the mean
TNF: tumour necrosis factor
