# Supplementary figures and images for: Neuroinflammation in the normal-appearing white matter (NAWM) of the multiple sclerosis brain causes abnormalities at the nodes of Ranvier
Source: PLoS Biol. 2020 Dec 14;18(12):e3001008. doi: 10.1371/journal.pbio.3001008 (PMC7769608; doi:10.1371/journal.pbio.3001008)

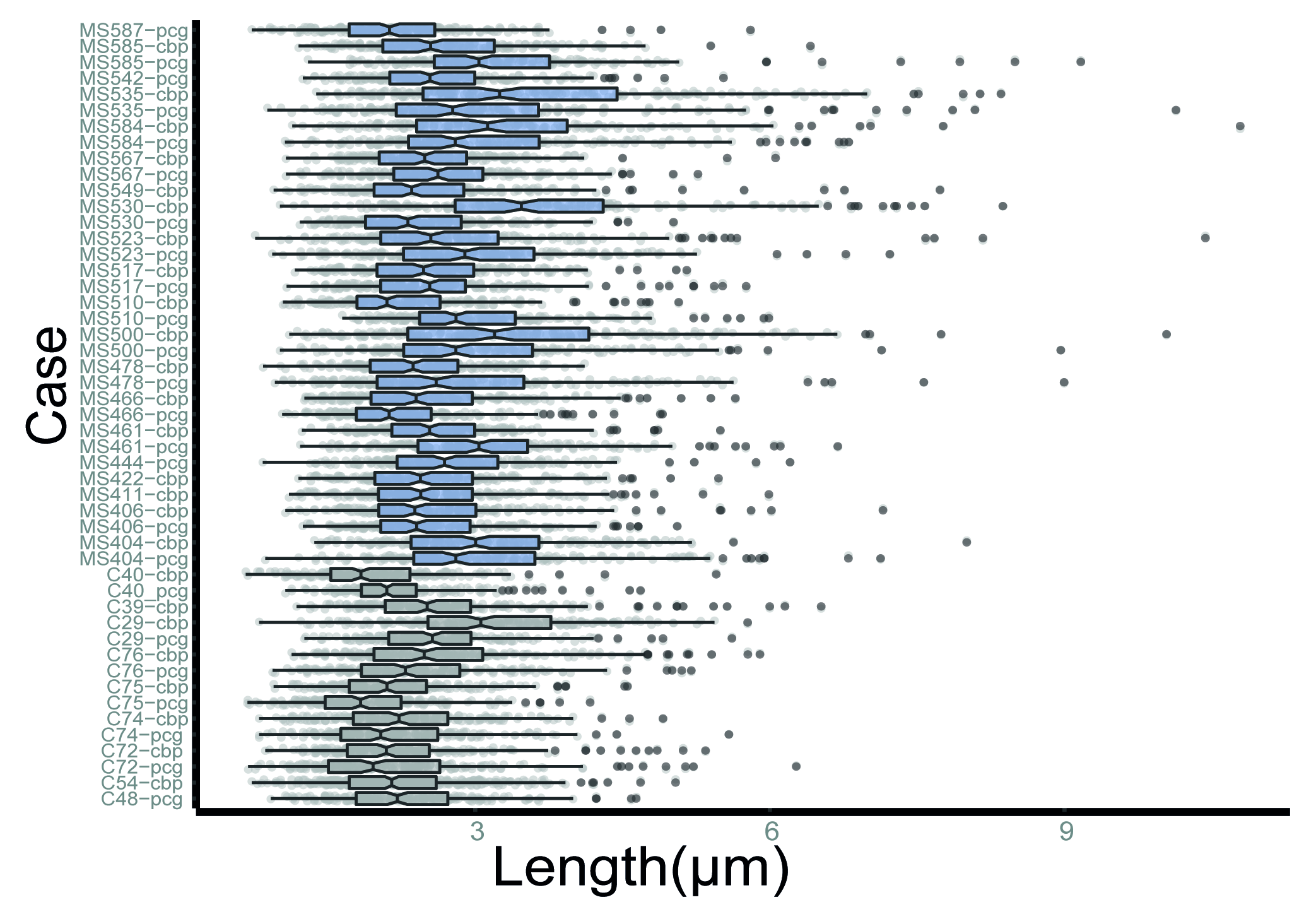

Supplement: S1 Fig — Box plots representing the distributions of paranodal length from NAWM MS and non-neurological control tissue per case. In the y axis, the indexes “cbp” correspond to the cerebral peduncle blocks, while the indexes “pcg” correspond to the precentral gyrus blocks. MS, multiple sclerosis; NAWM, normal-appearing white matter. Data and code to reproduce this figure can be found at: https://github.com/PatGal2020/PLOS_submission. (TIF) [file pbio.3001008.s001.tif]

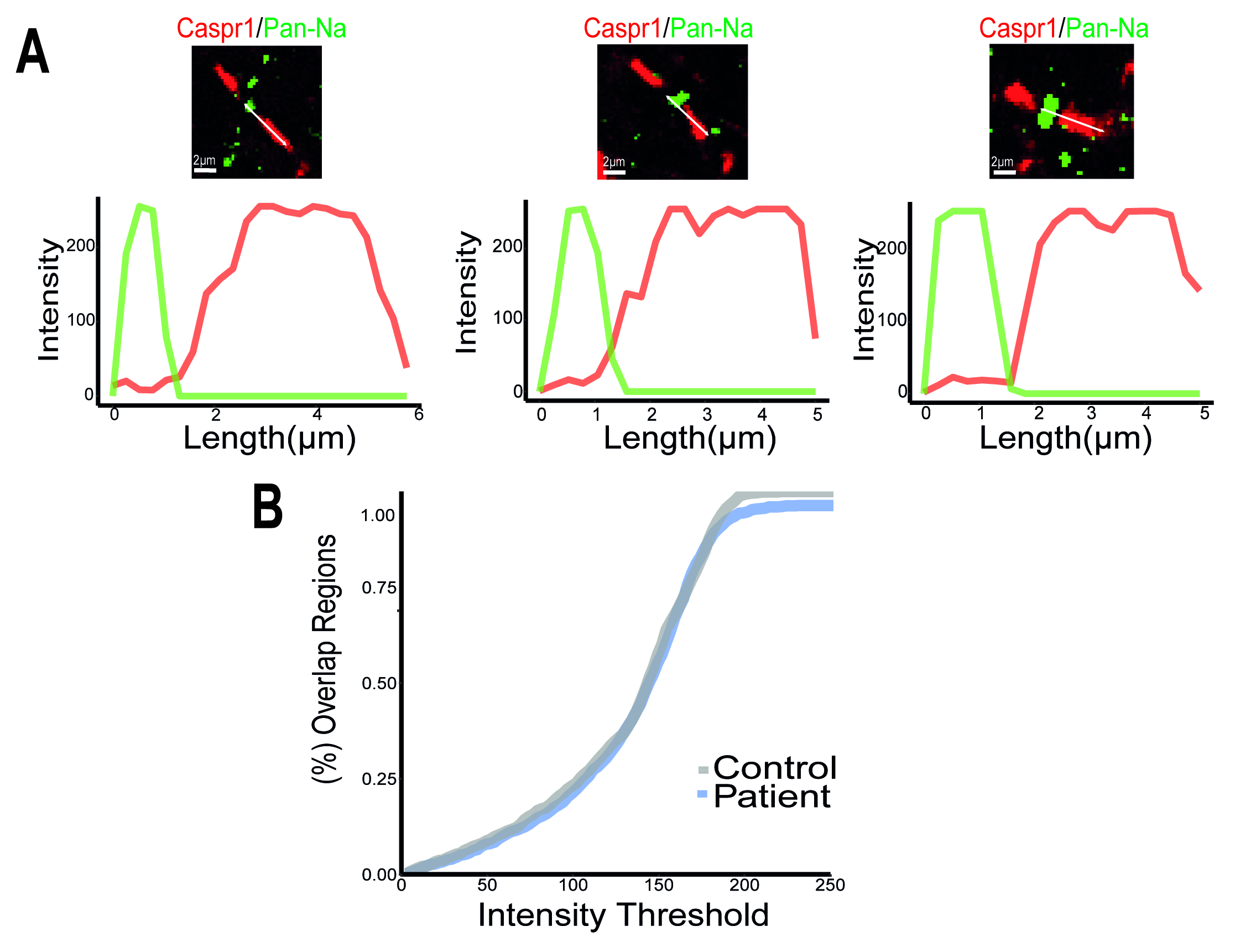

Supplement: S2 Fig — (A) Confocal images of a double immunofluorescence of Caspr1-stained paranode and nodal voltage-gated Nav channels with the RGB intensity profile of both immunofluorescence signals across the nodal and paranodal compartments. (B) Caspr1 signal was subtracted from Nav, and when the difference between them was smaller than a variable Intensity Threshold, that point was considered an overlapping region. For every threshold calculated, the proportion of overlapping regions was very similar in both groups. MS, multiple sclerosis; NAWM, normal-appearing white matter. Data and code to reproduce this figure can be found at: https://github.com/PatGal2020/PLOS_submission. (TIF) [file pbio.3001008.s002.tif]

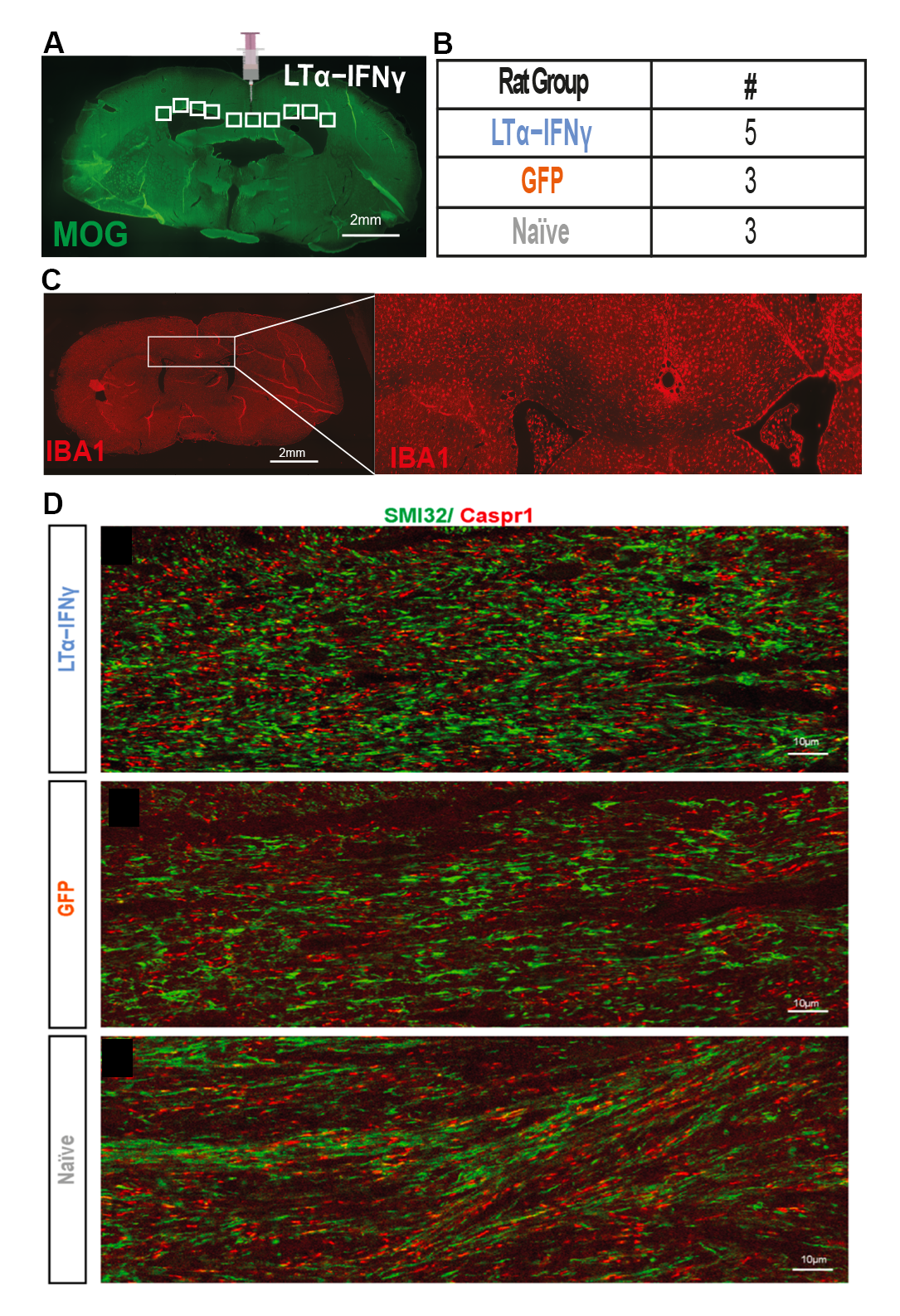

Supplement: S3 Fig — (A) Immunofluorescent image of a coronal rat section stained with MOG. Lentiviral vectors encoding LT-α and IFN-γ genes were injected into the subarachnoid space in the midline of the brain. The white rectangles are a representative of the 10 selected ROIs at the corpus callosum, cingulum, and external capsule. (B) Table of the number of animals used: 5 rats were injected with LTα/IFNγ, 3 rats with GFP, and 3 naives. (C) Immunofluorescent image of a coronal rat section stained with IBA1 and treated with LT-α and IFN-γ. (D) Caspr1-SMI32 immunofluorescence in LTα/IFNγ, GFP, and naive rat tissue. Confocal images of Caspr1-stained paranodes (red) and SMI32+ axons (green). GFP, green fluorescent protein; IFNγ, interferon-γ; LTα, lymphotoxin-α; MOG, myelin oligodendrocyte glycoprotein; ROI, region of interest. Data and code to reproduce this figure can be found at: https://github.com/PatGal2020/PLOS_submission. (TIF) [file pbio.3001008.s003.tif]

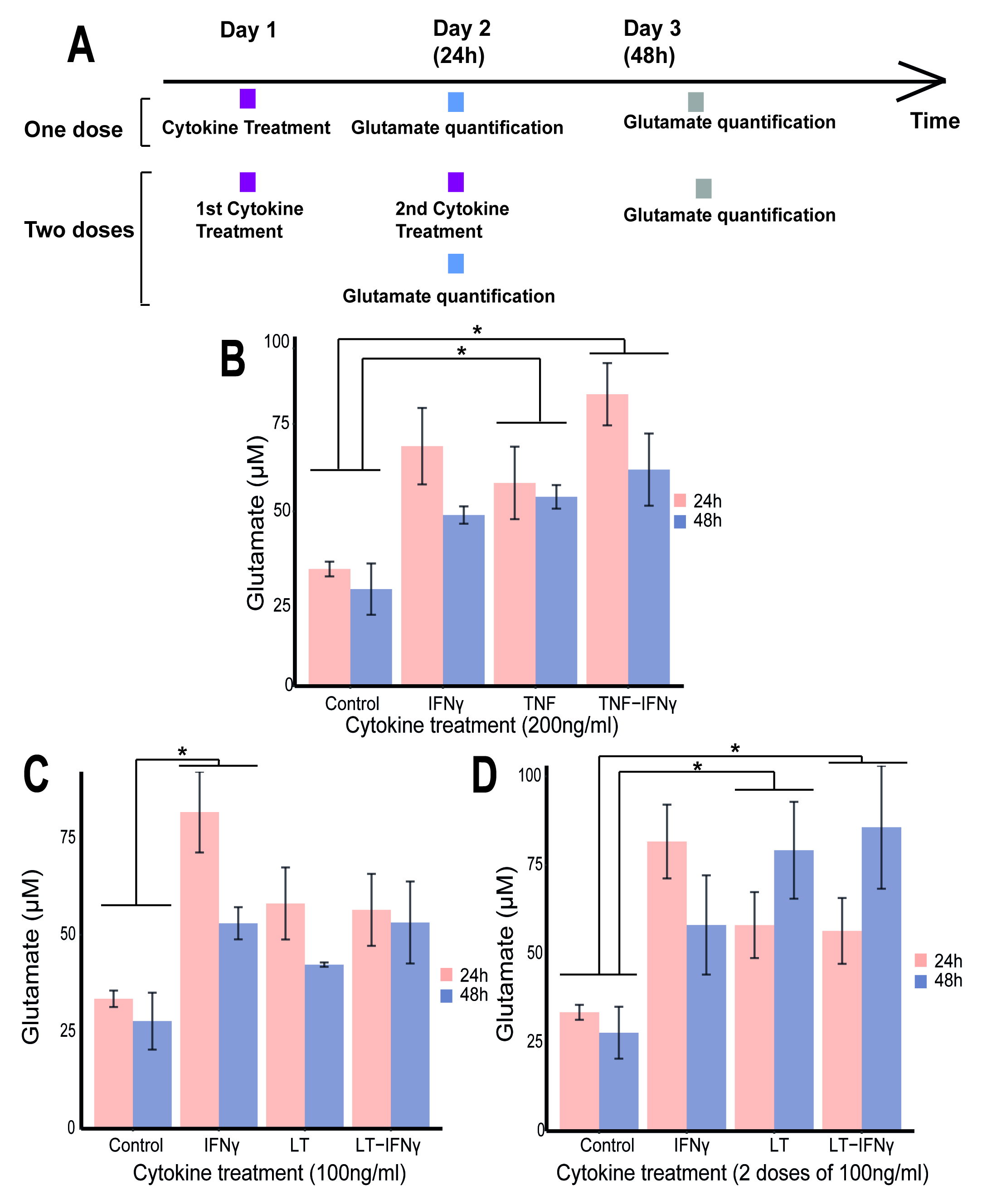

Supplement: S4 Fig — (A) Timeline diagram showing the timings of the experiments. Microglia were either treated with 1 dose of cytokines or 2 doses 24 h apart. The glutamate in the supernatant was analysed in both cases after 24 h and 48 h. (B) Mean ± SEM glutamate levels from replicates showing the statistical difference between controls and the cytokine treatments at different concentrations: 200 ng/ml (n = 3 Control, n = 3 TNF, n = 3 IFNγ, n = 3 TNF + IFNγ), (C) 100 ng/ml (n = 3 Control, n = 3 LTα, n = 3 IFNγ, n = 3 LTa + IFNγ), and (D) 2 acute treatments with 100 ng/ml (n = 3 Control, n = 3 LTα, n = 3 IFNγ, n = 3 LTα + IFNγ). Nonparametric Friedman test was performed across cytokine groups and timings and post hoc paired-wised Wilcoxon tests to compare groups (* p < 0.05, ** p < 0.01). IFNγ, interferon-γ; LTα, lymphotoxin-α; TNF, tumour necrosis factor. Data and code to reproduce this figure can be found at: https://github.com/PatGal2020/PLOS_submission. (TIF) [file pbio.3001008.s004.tif]

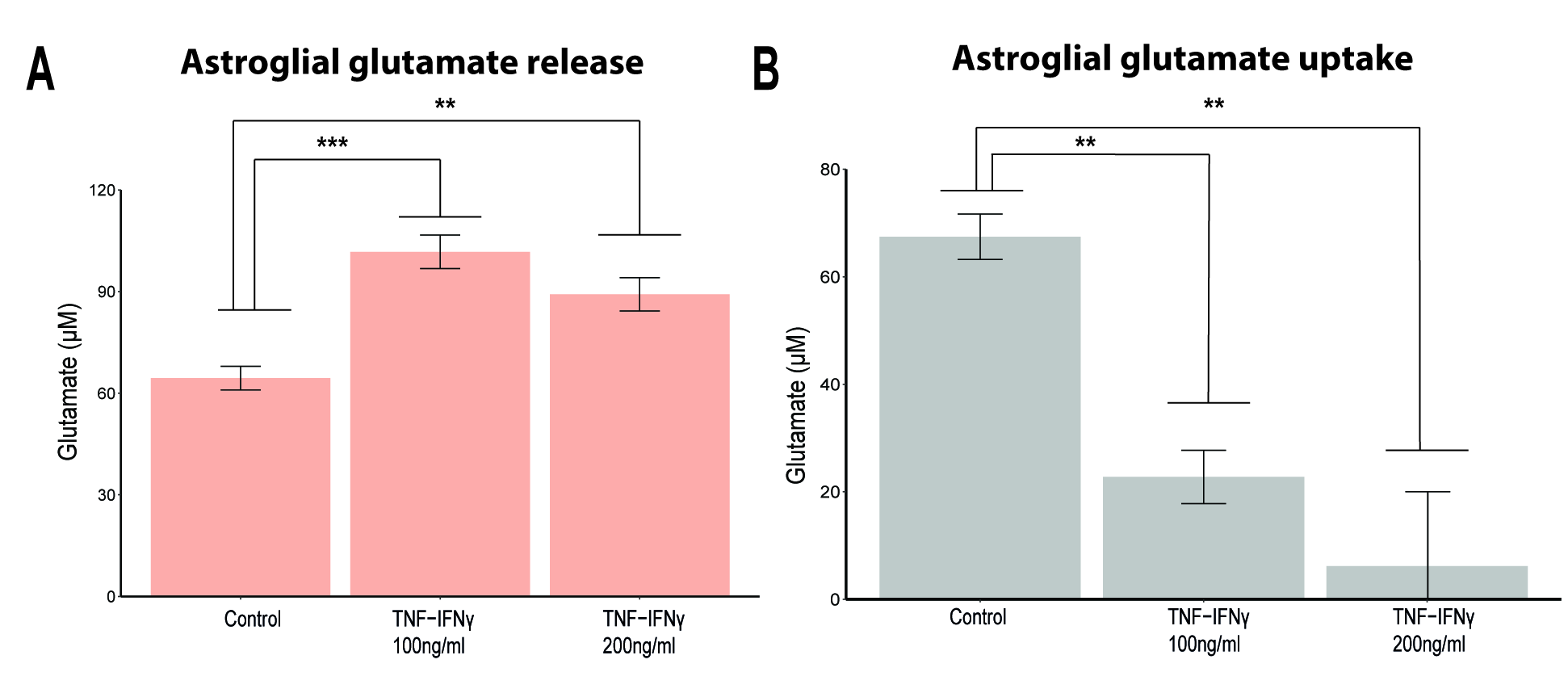

Supplement: S5 Fig — (A) Glutamate release by primary astrocyte cultures treated with TNF/IFNγ (100 ng/ml and 200 ng/ml) after 24 h. (B) Glutamate uptake by primary astrocyte cultures treated with TNF/IFNγ (100 ng/ml and 200 ng/ml) and 100 μM of glutamate after 24 h. Mean ± SEM for glutamate levels from replicates showing the statistical difference between controls and the cytokine treatments. Nonparametric Mann–Whitney test was performed across cytokine groups. IFNγ, interferon-γ; TNF, tumour necrosis factor. Data and code to reproduce this figure can be found at: https://github.com/PatGal2020/PLOS_submission. (TIF) [file pbio.3001008.s005.tif]

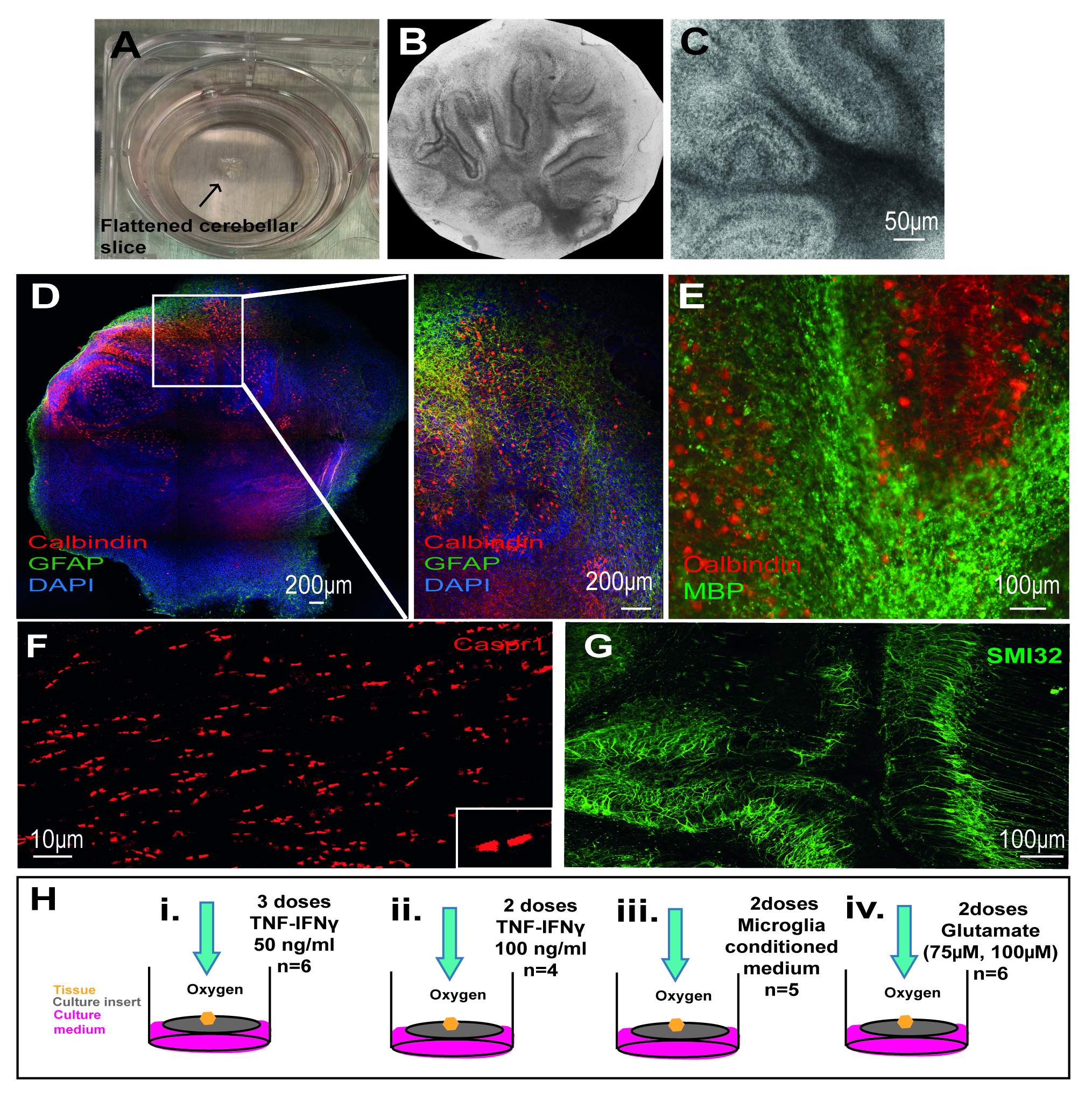

Supplement: S6 Fig — (A) Image of a live flattened cerebellar slice. The slices were cut at 400 μm thickness and after 8–10 DIV healthy slices flatten to approximately 100 μm. (B, C) Bright field images of cerebellar slices on culture inserts. (D) Confocal image of a cerebellar slice stained with antibodies against Calbindin+ for Purkinje cells and GFAP+ for astroglia. (E) Confocal image of a cerebellar slice stained with antibodies to MBP for myelin and Calbindin for Purkinje cells. (F) Confocal image of a cerebellar slice stained with Caspr1 antibodies. (G) Confocal image of a cerebellar slice stained with SMI32 antibodies. (H) Cerebellar slices were treated with the pro-inflammatory cytokines TNF/IFNγ (3 doses of 50 ng/ml (n = 3), 2 doses of 100 ng/ml (n = 4)), microglial-conditioned medium (2 doses of the medium from microglia treated with 2 acute doses of 100 ng/ml of TNF/IFNγ), and glutamate (2 doses of 75 mM or 100 mM). IFNγ, interferon-γ; MBP, myelin basic protein; TNF, tumour necrosis factor. Data and code to reproduce this figure can be found at: https://github.com/PatGal2020/PLOS_submission. (TIF) [file pbio.3001008.s006.tif]

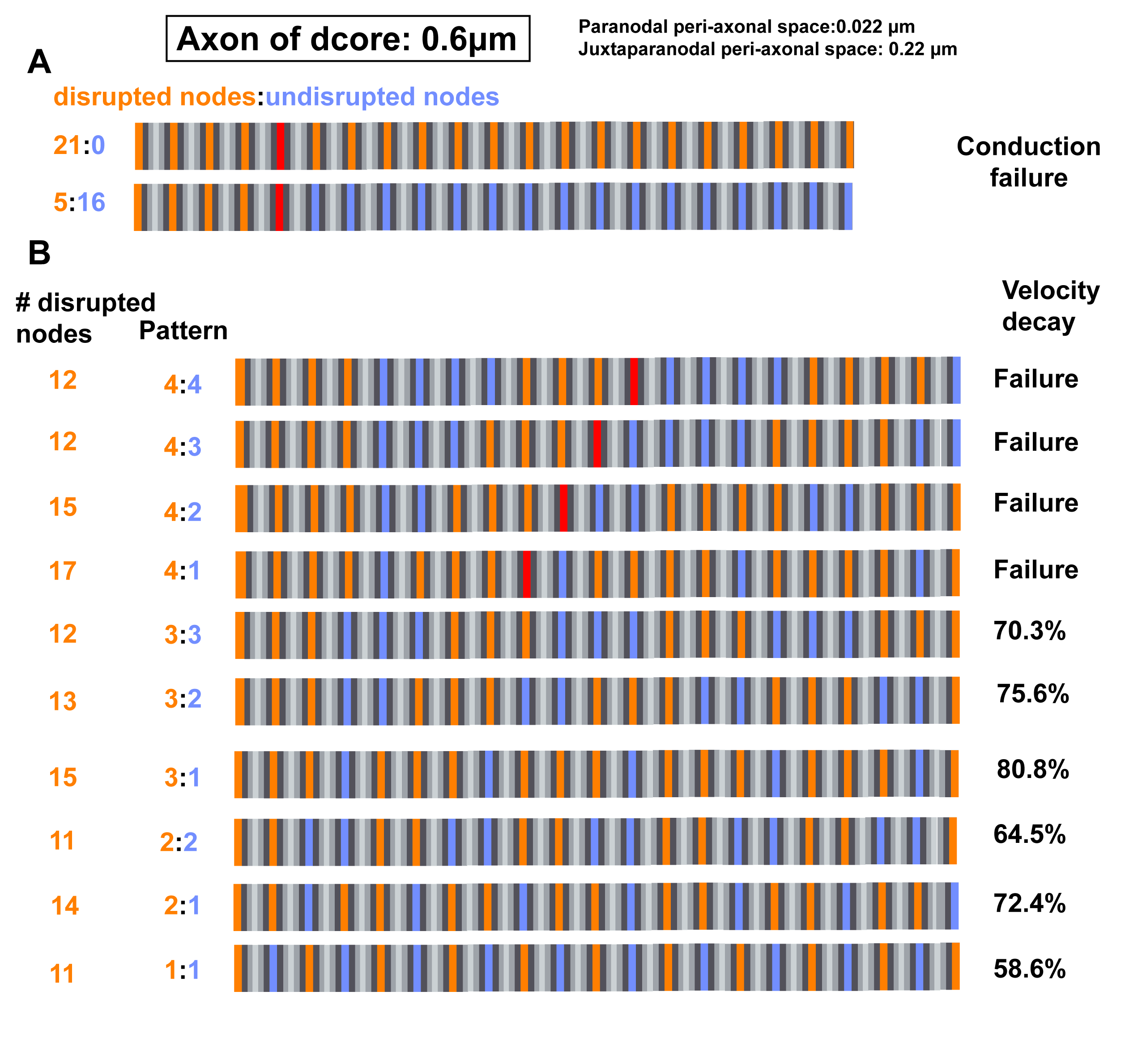

Supplement: S7 Fig — The difference in the proportion of disrupted paranodes within an axon of dcore of 0.6 μm can provoke conduction failure and a variable degree of velocity reduction. (A) In axon model of 0.6 μm core diameter, conduction failure occurred when 5 consecutive nodes were disrupted (orange), and the paranodal and juxtaparanodal peri-axonal space widths were increased up to 0.022 and 0.22 μm, respectively. (B) Velocity decay and conduction failure of this axon model under different patterns of disruption (orange means disrupted node, purple, healthy node, and red denotes conduction failure). (TIF) [file pbio.3001008.s007.tif]
