# Supplementary material for: Neuroinflammation in the normal-appearing white matter (NAWM) of the multiple sclerosis brain causes abnormalities at the nodes of Ranvier
Source: PLoS Biol. 2020 Dec 14;18(12):e3001008. doi: 10.1371/journal.pbio.3001008 (PMC7769608; doi:10.1371/journal.pbio.3001008)
Supplement: S1 Text — (A) Algorithm for the quantification of Caspr1-Kv or Caspr1-Na overlapping signals. (B) Code for the computational model generated in NEURON. (DOCX) [file pbio.3001008.s009.docx]

Supplementary 1 Code:

**A.**

**Algorithm for the quantification of Caspr1-Kv or**

**Caspr1-Na overlapping signals**

rm(list =ls())

gc()

overall.wd = "C:/Users/pg2015/Desktop/Measurements/patients_K"

setwd(overall.wd)

file.list1 = list.files(path = ".") # list of all files

file.len1 = length(file.list1)

library(ggplot2)

total.data1 = c()

for(f in 1:file.len1){

print(f)

# get all files:

setwd(paste0(overall.wd,sep = "//",file.list1[1]))

folder.list1 = list.files(path = ".") # list of all files

folder.len1 = length(folder.list1)

for(i in 1:folder.len1){

cat("i =", i, "\n")

data1 = read.csv(folder.list1[i],header = TRUE)

clean.data1 = data.frame('X0' = data1$X0,'Y0'= data1$Y0,'Y1'= data1$Y1,'Y2'= data1$Y2)

total.data1 = rbind(clean.data1, total.data1)

}

}

total.data1=total.data1

#establishing the overlap method

minus1 = abs(total.data1$Y0 - total.data1$Y2)

thresholdseq1=seq(0, max(max(total.data1$Y0),max(total.data1$Y2)),1.5)

cushion1=10000

overlaps1 = c()

tmin1 = 75

9

for (j in thresholdseq1){

# for each threshold do:

pair.store1 = c() # store pairs of start/end here

count = 1 # start looking from first entry onwards

ovlps1 = length(minus1[minus1 <= j & total.data1$Y0 > tmin1 & total.data1$Y2 > tmin1])/length(

minus1)

overlaps1 = c(overlaps1, ovlps1)

}

**B.**

**NEURON computational model**

proc model_globels() {

celsius=37

v_init=-80 //mV//

dt=0.001 //ms//

tstop=10

//Intracellular stimuluation parameters//

istim=2

delay=1

pw=0.1

//topological parameters//

axnodes=21

axparanodes=40

axjuxtaparanodes=40

axinternodes=120

axontotal=221

//morphological parameters//

fiberD=0.5//choose from 0.5, 0.8, 1.1, 1.3, 1.6, 1.8, 3.5 //um

paralength1=2.3 //um

nodelength=1.0 //um

space_p0=0.002//um

space_p1=0.002//um

space_p2=0.02 //um

space_i=0.02 //um

//electrical parameters//

rhoa=70 //Ohm-cm//

axon_capacitance=1 //uF/cm2//

myelin_rhoa=1000 //Ohm-cm2//

myelin_capacitance=0.1 //uF/cm2//

10

}

model_globels ()

// the units of Rpn are Ohm.cm and not cm^2.

proc dependent_var() {

if (fiberD==0.8) {axonD=0.6 nodeD=0.6 paraD1=0.6 paraD2=0.6 paralength2=2.58*fiberD + 19.6

node_node_L=117.52+30.47*fiberD interlength=(node_node_L-2*(paralength2+paralength1))/6 nl=6

thick_myelin=0.09 }

if (fiberD==1.1) {axonD=0.8 nodeD=0.8 paraD1=0.8 paraD2=0.8 paralength2=2.58*fiberD+19.6

node_node_L=117.52+30.47*fiberD interlength=(node_node_L-2*(paralength2+paralength1))/6 nl=8

thick_myelin=0.12}

if (fiberD==1.3) {axonD=1 nodeD=1 paraD1=1 paraD2=1 paralength2=2.58*fiberD+19.6

node_node_L=117.52+30.47*fiberD interlength=(node_node_L-2*(paralength2+paralength1))/6 nl=10

thick_myelin=0.15}

if (fiberD==1.6) {axonD=1.2 nodeD=1.2 paraD1=1.2 paraD2=1.2 paralength2=2.58*fiberD+19.6

node_node_L=117.52+30.47*fiberD interlength=(node_node_L-2*(paralength2+paralength1))/6 nl=12

thick_myelin=0.18}

if (fiberD==1.8) {axonD=1.4 nodeD=1.4 paraD1=1.4 paraD2=1.4 paralength2=2.58*fiberD+19.6

node_node_L=117.52+30.47*fiberD interlength=(node_node_L-2*(paralength2+paralength1))/6 nl=13

thick_myelin=0.2}

if (fiberD==3.5) {axonD=2.7 nodeD=2.7 paraD1=2.7 paraD2=2.7 paralength2=2.58*fiberD+19.6

node_node_L=117.52+30.47*fiberD interlength=(node_node_L-2*(paralength2+paralength1))/6 nl=26

thick_myelin=0.4}

if (fiberD==0.5) {axonD=0.4 nodeD=0.4 paraD1=0.4 paraD2=0.4 paralength2=2.58*fiberD+19.6

node_node_L=117.52+30.47*fiberD interlength=(node_node_L-2*(paralength2+paralength1))/6 nl=3

thick_myelin=0.04}

//axon and myelin areas

axon_xarea=PI*(axonD/2)^2 //um2

node_surface_area=PI*nodelength*axonD //um2

para_surface_area=PI*paralength1*axonD //um2

juxta_surface_area=PI*paralength2*axonD //um2

inter_surface_area=PI*interlength*axonD //um2

node_periaxonal_xarea=(PI*(((axonD/2)+space_p0)^2))-axon_xarea //

um2

para_periaxonal_xarea=((PI*(((axonD/2)+space_p1)^2))-axon_xarea)+(170*(10^-6)*(nl-1)) //

11

um2 170nm2 is the third pathway xarea in the the paranodal space (Mierwa et al 2009)

juxta_periaxonal_xarea=(PI*(((axonD/2)+space_p2)^2))-axon_xarea //

um2

inter_periaxonal_xarea=(PI*(((axonD/2)+space_i)^2))-axon_xarea //

um2

para_myelin_surface_area=PI*(axonD+2*space_p1+thick_myelin)*paralength1 //

um2

juxta_myelin_surface_area=PI*(axonD+2*space_p2+thick_myelin)*paralength2 //

um2

inter_myelin_surface_area=PI*(axonD+2*space_i+thick_myelin)*interlength //um2

//axon resistance and capacitance

node_axon_resistance=(rhoa*nodelength)/axon_xarea //

Ohm cm/um

para_axon_resistance=(rhoa*paralength1)/axon_xarea //

Ohm cm/um

juxta_axon_resistance=(rhoa*paralength2)/axon_xarea //

Ohm cm/um

inter_axon_resistance=(rhoa*interlength)/axon_xarea //

Ohm cm/um

node_axon_capacitance=axon_capacitance*node_surface_area //uF

um2/cm2

para_axon_capacitance=axon_capacitance*para_surface_area //uF

um2/cm2

juxta_axon_capacitance=axon_capacitance*juxta_surface_area //uF

um2/cm2

inter_axon_capacitance=axon_capacitance*inter_surface_area //uF

um2/cm2

//Myelin resistance and capacitance

para_myelin_resistance=(myelin_rhoa*nl*2)/para_myelin_surface_area //

Ohm cm2/um2

juxta_myelin_resistance=(myelin_rhoa*nl*2)/juxta_myelin_surface_area //Ohm

cm2/um2

inter_myelin_resistance=(myelin_rhoa*nl*2)/inter_myelin_surface_area //Ohm

12

cm2/um2

para_myelin_capacitance=(myelin_capacitance*nl*2)/para_myelin_surface_area //uF

um2/cm2

juxta_myelin_capacitance=(myelin_capacitance*nl*2)/juxta_myelin_surface_area //uF

um2/cm2

inter_myelin_capacitance=(myelin_capacitance*nl*2)/inter_myelin_surface_area //uF

um2/cm2

//periaxonal resistance

Rpn0=(rhoa/(node_periaxonal_xarea*10^-8))*0.000001 //

Mohm/cm

Rpn1=(rhoa/(para_periaxonal_xarea*10^-8))*0.000001 //

Mohm/cm

Rpn2=(rhoa/(juxta_periaxonal_xarea*10^-8))*0.000001 //

Mohm/cm

Rpx=(rhoa/(inter_periaxonal_xarea*10^-8))*0.000001 //

Mohm/cm

}

dependent_var()

objectvar stim

create nodes[axnodes], paranodes[axparanodes], juxtaparanodes[axjuxtaparanodes], internodes[

axinternodes]

access nodes[0] //default value when plotting

proc initialize(){

for i=0,axnodes-1 {

nodes[i]{

nseg=1

diam=nodeD

L=nodelength

Ra=rhoa // 70 Ohm.cm

cm=1

insert axnode

13

insert extracellular xraxial=Rpn0 xg=10e9 xc=0 //xg(S/cm2) xc(uF/cm2)

}

}

for i=0, axparanodes-1 {

paranodes[i]{

nseg=1

diam=paraD1

L=paralength1

Ra=rhoa

cm=1

insert pas

g_pas=0.0005

e_pas=-80

insert extracellular xraxial=Rpn1 xg=1/(para_myelin_resistance*

para_surface_area) xc=(para_myelin_capacitance/para_surface_area)

}

}

for i=0, axjuxtaparanodes-1 {

juxtaparanodes[i]{

nseg=1

diam=paraD2

L=paralength2

Ra=rhoa

cm=1

insert axjuxtaparanode

insert pas

g_pas=0.005

e_pas=-80

insert extracellular xraxial=Rpn2 xg=1/(juxta_myelin_resistance*

juxta_surface_area) xc=(juxta_myelin_capacitance/juxta_surface_area)

}

}

for i=0, axinternodes-1 {

internodes[i]{

nseg=1

diam=axonD

L=interlength

Ra=rhoa

14

cm=1

insert pas

g_pas=0.005

e_pas=-80

insert extracellular xraxial=Rpx xg=1/(inter_myelin_resistance*

inter_surface_area) xc=(inter_myelin_capacitance/inter_surface_area)

}

}

for i=0, axnodes-2 {

connect paranodes[2*i](0), nodes[i](1)

connect juxtaparanodes[2*i](0), paranodes[2*i](1)

connect internodes[6*i](0), juxtaparanodes[2*i](1)

connect internodes[6*i+1](0), internodes[6*i](1)

connect internodes[6*i+2](0), internodes[6*i+1](1)

connect internodes[6*i+3](0), internodes[6*i+2](1)

connect internodes[6*i+4](0), internodes[6*i+3](1)

connect internodes[6*i+5](0), internodes[6*i+4](1)

connect juxtaparanodes[2*i+1](0), internodes[6*i+5](1)

connect paranodes[2*i+1](0), juxtaparanodes[2*i+1](1)

connect nodes[i+1](0), paranodes[2*i+1](1)

}

finitialize(v_init)

fcurrent()

}

initialize()

//intracellular stimulus//

proc stimul() {

nodes[0]{

stim=new IClamp()

stim.loc(.5)

stim.del=delay

stim.dur=pw

stim.amp=istim

}

}

stimul()

15

xpanel("Stimulus parameters")

xvalue("Stimulus Amplitude (nA)", "istim", 1, "stimul()", 1)

xvalue("Pulse Duration (ms)", "pw", 1)

xvalue("Onset Delay (ms)", "delay", 1)

xpanel(100,100)

//recording and saving data from voltage node4

objref rect, recv

rect = new Vector()

recv = new Vector()

recv.record(&nodes[4].v(0.5))

rect.record(&t)

run()

recv.printf()

objref savdata

savdata = new File()

savdata.wopen("node4_2.7.txt")

savdata.printf("t node[4].v(0.5)\n")

for i=0,rect.size()-1 {

savdata.printf("%g %g\n", rect.x(i), recv.x(i))

}

savdata.close()

//recording and saving data from voltage node16

16

objref recvtwo, recttwo

recvtwo = new Vector()

recttwo=new Vector()

recvtwo.record(&nodes[16].v(0.5))

recttwo.record(&t)

run()

recvtwo.printf()

objref savdatatwo

savdatatwo = new File()

savdatatwo.wopen("node16_2.7.txt")

savdatatwo.printf("t node[16].v(0.5)\n")

for i=0,recttwo.size()-1 {

savdatatwo.printf("%g %g\n", recttwo.x(i), recvtwo.x(i))

}

savdatatwo.close()

//recording current node 4

objref recti, reci

reci = new Vector()

recti=new Vector()

reci.record(&nodes[4].ina_axnode(0.5))

recti.record(&t)

run()

reci.printf()

objref savdatai

savdatai = new File()

savdatai.wopen("node4_current_2.7.txt")

savdatai.printf("t node[4].ina_axnode(0.5)\n")

17

for i=0,recti.size()-1 {

savdatai.printf("%g %g\n", recti.x(i), reci.x(i))

}

savdatai.close()

18
